# HIDF: Integrating Tree‐Structured scRNA‐seq Heterogeneity for Hierarchical Deconvolution of Spatial Transcriptomics

**DOI:** 10.1002/advs.202514073

**Published:** 2025-12-12

**Authors:** Zhiyi Zou, Yuting Bai, Bo Wang, Wanwan Shi, Xiao Liang, Jiawei Luo

**Affiliations:** ^1^ College of Computer Science and Electronic Engineering Hunan University Changsha 410082 China

**Keywords:** cell type deconvolution, single‐cell RNA sequencing, spatial transcriptomic

## Abstract

The limited spatial resolution of mainstream spatial transcriptomic technologies captures transcriptomic mixtures from multiple cells per spot, obscuring crucial single‐cell information. While numerous methods leverage single‐cell RNA sequencing references to infer cellular composition from ST data, they primarily rely on fixed cell type labels, overlooking the intrinsic hierarchical heterogeneity (subtypes within broad types) of cellular populations and its association with spatial organization. To address this limitation, HIDF, a Hierarchical Iterative Deconvolution Framework is proposed. HIDF progressively resolves cellular heterogeneity from coarse to fine granularity, it employs a hierarchical iterative optimization mechanism guided by the cluster‐tree to recover single‐cell spatial distributions. This process is further stabilized and enhanced by incorporating dual regularization constraints (spatial neighborhood and cross‐level regularization). Comprehensive benchmarking demonstrates that HIDF outperforms existing methods on simulated and real tissue datasets. In addition, HIDF not only reveals cell type distributions consistent with known tissue functions but also uncovers spatially heterogeneous patterns of cell subtypes undetectable by conventional methods.

## Graphical abstract

The prevailing neglect of cellular hierarchies in current spatial transcriptomics deconvolution often obscures cellular heterogeneity and impedes the identification of fine‐grained subtypes. To address this issue, HIDF employs a cluster‐tree and dual regularization to systematically model cellular hierarchical structures. This approach significantly improves deconvolution accuracy, achieves precise subtype‐level resolution, and provides a foundation for a deeper understanding of tissue spatial organization.

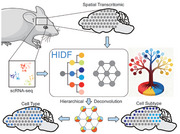

## 1 Introduction

Advances in Spatial Transcriptomics (ST) enable high‐throughput analysis of gene expression profiles within the spatial context of tissues,^[^ ^1^ ^]^ providing unprecedented insights into the distribution of cellular heterogeneity within complex structures, such as brain organization,^[^ ^2^, ^3^ ^]^ tumor microenvironments,^[^ ^4^, ^5^ ^]^ and immune niches.^[^ ^6^ ^]^ However, a fundamental limitation of current mainstream ST technologies (e.g., 10x Visium) is their inherent spatial resolution constraint. Their capture spot size (55 µ*m*) is larger than the size of a single cell (5–10 µ*m*).^[^ ^7^ ^]^ Consequently, each spot inherently captures transcriptomic signals representing a mixture of multiple potentially heterogeneous cells. This signal averaging significantly obscures critical single‐cell resolution information, including the precise spatial localization of fine cell types/subtypes. Although emerging imaging‐based ST technologies offer single‐cell or even subcellular resolution(e.g., seqFISH,^[^ ^8^ ^]^ MERFISH,^[^ ^9^ ^]^ STARmap^[^ ^10^ ^]^), their limited gene detection capacity (typically only hundreds of preselected genes) hinders systematic discovery at the whole‐transcriptome scale and the exploration of biological phenomena.^[^ ^7^, ^11^ ^]^ Therefore, a core computational challenge remains: accurately recovering single‐cell resolution spatial information (e.g., cell‐type composition and distribution) from low‐resolution, whole‐transcriptome ST data.

Single‐cell RNA sequencing (scRNA‐seq) generates cellular reference profiles at single‐cell resolution through tissue dissociation, thus providing the essential cell‐type‐specific expression data required to deconvolve mixed signals within spots. Consequently, numerous deconvolution methods leveraging annotated scRNA‐seq references have emerged in recent years to decipher cellular composition from ST data. These approaches can be broadly classified into three categories: 1) Pseudo‐spot methods based on simulation strategies (e.g., SPACEL,^[^ ^12^ ^]^ SpatialPrompt^[^ ^13^ ^]^). These methods construct synthetic pseudo‐spots using single‐cell reference data, and use them to train deconvolution models for inferring the cell type proportions in real spatial transcriptomic data. The limitation of such approaches lies in the potential diversity between pseudo‐spots and real tissues, which may affect the accuracy of deconvolution results. Additionally, the output of these methods is essentially the estimation of the proportion of cell types at the spot level, which can not achieve the spatial deconvolution at single cell resolution. 2) Coarse‐grained models based on linear weighting (e.g., CARD,^[^ ^14^ ^]^ RCTD,^[^ ^15^ ^]^ Cell2location^[^ ^16^ ^]^). These methods generate average expression vectors as references by aggregating single‐cell expressions within the same cell type. They assume that the expression of a spot is a linear weighted combination of expression profiles of these cell types, and accordingly infer the cell type distribution. However, the average expression vector of a cell type is difficult to fully represent the multiple subtypes that may exist within these cells, such methods ignore the transcriptional diversity within cell types. Notably, the deconvolution results remain at the level of the proportion of cell types in the spot, which essentially cannot analyze the single‐cell spatial deconvolution. 3) Fine‐grained methods based on spatial mapping (e.g., Tangram,^[^ ^17^ ^]^ GraphST^[^ ^18^ ^]^ Redeconv^[^ ^19^ ^]^). These methods are aim to construct a direct mapping matrix from single cell to spots, which can, in theory, realize spatial deconvolution at the cellular level. However, such methods are often trapped in the complexity of high‐dimensional optimization problems, prone to fall into local optimal solutions. Which may lead to the difficulty to achieve accurate deconvolution of single‐cell resolution stably and reliably.

While the aforementioned three categories of methods have achieved notable successes in deconvolution tasks, they share a fundamental limitation: their reliance on fixed cell type labels derived from reference datasets as absolute classification criteria. This approach inherently overlooks the intrinsic heterogeneity present within cellular populations, particularly the differentiation of subtypes within broader cell types. This hierarchical heterogeneity can be effectively captured through the construction of a tree structure via unsupervised hierarchical clustering.^[^ ^20^, ^21^ ^]^ The branching patterns within this tree not only represent potential cell developmental trajectories but are also frequently associated with spatial distribution patterns, as exemplified by the spatially stratified organization of cortical neuronal subtypes.^[^ ^22^, ^23^ ^]^ However, most current methodologies, constrained by their dependence on discrete, fixed cell type labels, fail to adequately leverage this hierarchical information. Although scMoE^[^ ^24^ ^]^ attempts to leverage hierarchical information from scRNA‐seq data for cell type deconvolution, it is not specifically designed for spatial transcriptomics. It fails to adequately account for batch effects between reference and spatial datasets, does not incorporate spatial information, and lacks single‐cell resolution. Consequently, existing methods still struggle to resolve the precise spatial distribution of cell types within complex tissues.

To address this limitation, this study proposes a Hierarchical Iterative Deconvolution Framework (HIDF). HIDF leverages the complementary characteristics of scRNA‐seq and spatial ST data to progressively resolve cellular heterogeneity—from broad cell types to fine‐grained subtypes. The innovations of HIDF are as follows: 1) Hierarchical tree construction. Generating a hierarchical clustering tree of scRNA‐seq to form a multi‐level gene reference atlas. 2) Hierarchical iterative deconvolution mechanism. Hierarchical reconstruction is realized by optimizing the spatial mapping matrix to resolve cell type composition. It resolves cell‐type composition by optimizing spatial mapping matrix, then decomposes inferred proportions guided by cluster‐tree topology to iteratively initialize fine‐grained spatial distributions for progressive refinement. 3) Dual regularization constraints. Combining spatial regularization and cross‐level consistency constraints to ensure the accuracy and stability of spatial deconvolution at single‐cell level. To demonstrate the unique advantages of HIDF, we elaborate on it in the supplementary text from four aspects: model complexity, computational cost, interpretability, and input requirements.

In three simulated datasets, HIDF not only demonstrated the accuracy of its cell type deconvolution, but also successfully addressed the spatial heterogeneity of cell subtypes. In real mouse brain, human breast cancer, and mouse thymus tissues, it further identified cell type distributions consistent with tissue functions, revealed spatial differential distributions of cell subtypes those traditional methods failed to capture.

## 2 Results

### 2.1 Overview of HIDF

**Figure 1:**
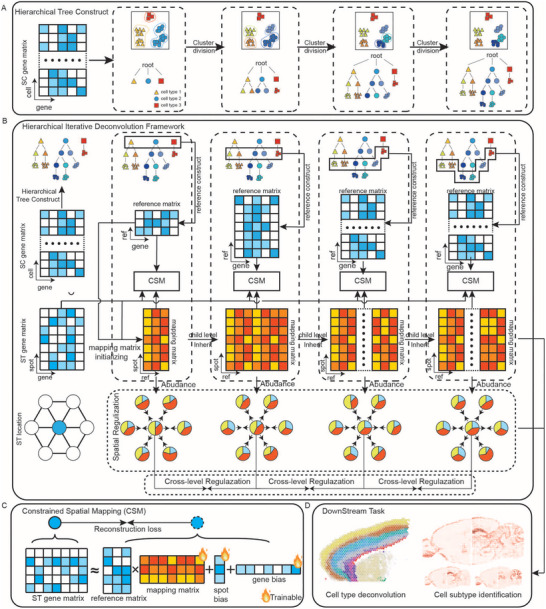
Overview of HIDF. A) Hierarchical tree construction: Built from scRNA‐seq reference data via top‐down cluster division, the tree organizes cell types into progressively finer subtypes down to individual cells. B) Hierarchical iterative deconvolution: Using the tree as reference, an iterative deconvolution progressively refines the SC‐to‐ST mapping matrix from coarse to fine grains. Optimization occurs level‐by‐level by minimizing a reconstruction loss constrained by spatial mapping. Upon convergence, the optimized matrix initializes the next level. During traning process, dual regularization is applied: cross‐level (ensuring consistency between levels) and spatial (promoting similarity in composition between adjacent spots). C) Constrained spatial mapping: ST data is reconstructed from single‐cell reference data via the mapping matrix. Batch effects are modeled using two additional bias parameters, with cosine similarity as the reconstruction loss function. D) Downstream tasks: Using the mapping matrix and annotated scRNA‐seq cell types, HIDF resolves cellular composition of ST spots. Its hierarchical approach reveals specific spatial distribution patterns of cell subtypes.

HIDF is a Hierarchical Iterative Deconvolution Framework to resolve cell type composition and spatial distribution from ST data. As shown in **Figure** **1**A, HIDF first constructs a hierarchical tree based on a scRNA‐seq reference dataset, establishing a multi‐level representation with nodes ranging from cell types to subtypes and single cells. Building upon this tree, HIDF employs a top‐down iterative optimization strategy (Figure 1B). It initiates at the cell type level, calculating average gene expression vectors as reference cell prototypes. Deconvolution is then performed by optimizing a constrained spatial mapping model using a cosine similarity reconstruction loss (Figure 1C). Upon convergence at a given level, the framework descends to its child nodes. It initializes the child‐level mapping weights using the parent‐level weights and computes new reference prototypes based on the child cell clusters before resuming optimization. This process iterates recursively down to the cellular level, progressively enhancing resolution. To improve robustness, dual regularization constraints are introduced: a cross‐level mean squared error loss enforces consistency in estimated cell type abundance between parent and child levels, while a spatial regularization loss, utilizing a dynamic memory bank storing historical abundances and a spatial neighborhood graph, preserves compositional continuity between adjacent spots. The entire framework is trained hierarchically, starting from the coarsest cell type level and optimizing layer by layer until all single‐cell reference data are incorporated. By integrating the trained mapping matrix with scRNA‐seq cell type information, this method can deconvolve ST data to resolve their cellular composition. Building on the hierarchical iterative deconvolution framework, HIDF further identifies spatial distribution patterns of cellular subtypes (Figure 1D).

### 2.2 Benchmark Experiments of Simulated Datasets

Due to the unknown true cell type abundances in real low‐resolution ST data, we assessed the deconvolution performance of HIDF and other methods using simulated datasets with known cell type proportions. We evaluated the deconvolution performance of HIDF on two simulated datasets of MERFISH and seqFISH.^[^ ^25^ ^]^ These datasets were generated by simulating low‐resolution spatial transcriptomic data based on the labeled single‐cell resolution ST data. We benchmarked HIDF against other methods on MERFISH (20/50/100 µ*m* resolution) and seqFISH (3,000/6,000/10,000 genes) datasets using Root Mean Square Error (RMSE). Lower RMSE values indicate higher deconvolution accuracy.

**Figure 2:**
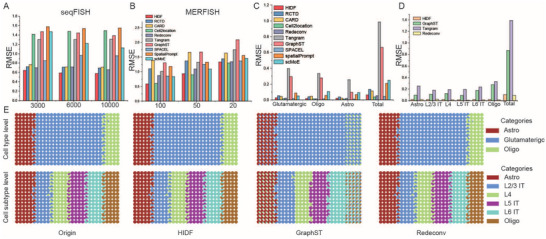
Benchmark experiments on simulated datasets. A,B) Bar plots display the RMSE per cell type and the total RMSE in seqFISH (gene numbers: 3000, 6000, 10000) and MERFISH (resolutions: 20, 50, 100 µ*m*) simulated datasets. C) Bar plots display the RMSE per cell type and the total RMSE in hierarchical simulated dataset. D) Bar plots display the RMSE per cell subtype and the total RMSE in hierarchical simulated dataset. E) Scatter pie plots show the spatial distribution of cell types and their subtypes on the hierarchical simulated dataset, comparing the deconvolution results of HIDF, GraphST, and Tangram with the ground truth of cell composition.

HIDF exhibited superior performance, as detailed below: 1) For the seqFISH dataset, across all gene expression scenarios (3,000/6,000/10,000 genes), HIDF achieved the lowest RMSE values (0.64, 0.59, 0.58; **Figure** **2**A). Compared to RCTD—the second‐best method—HIDF showed improvements of 11.1%, 16.9%, and 17.1%(RCTD's RMSE: 0.72, 0.71, 0.70; Tables S1–S3, Supporting Information).

2) In the MERFISH dataset, HIDF showed the best performance at 100 µ*m* resolution and the second‐best performance at 20 and 50 µ*m* (Figure 2B, and Tables S4–S6, Supporting Information), likely because HIDF more effectively captured the spatial continuity of cell distribution in low‐resolution data. Notably, the best method Cell2location at 20 and 50 µ*m*, achieved poor performance in the seqFISH dataset. RMSE ranked second or third worst. This highlights its methodological limitations. Although scMoE utilizes the hierarchical structure in single‐cell transcriptomic data, it demonstrates poor performance on simulated datasets. In conclusion, each cell type in these two simulated datasets, HIDF achieved the best or second‐best performance.

To further test HIDF's ability to dissect spatial heterogeneity, we built a hierarchical simulated dataset from mouse cerebral cortex scRNA‐seq data.^[^ ^26^ ^]^ The hierarchical simulated dataset consists of three basic cell types, astrocyte, glutamatergic, and oligodendrocyte. It contains four glutamatergic neuronal subtypes (L2/3, L4, L5, L6) that exhibit spatial hierarchy, which serves as a benchmark for validation. The specific simulation process of this dataset can be found in the Supporting Information.

Other methods (CARD, RCTD, Cell2location, SPACEL) accurately identified the three basic types but failed to resolve spatial heterogeneity among glutamatergic subtypes(Figure 2C). For Tangram and GraphST, though they captured subtype differences via cell‐spot mapping matrices, their RMSE values revealed accuracy deficiencies in deconvolution results. HIDF, by integrating hierarchical information from scRNA‐seq data, maintained basic cell type deconvolution accuracy (RMSE = 0.06) while successfully identifying the spatial distribution of glutamatergic subtypes. Redeconv achieved performance comparable to HIDF on this dataset (RMSE = 0.05).

The spatial distribution of glutamatergic sub‐clusters revealed key patterns. Glutamatergic subtypes resolved by HIDF exhibits regional enrichment. Notably, their distributions were consistent with those of the corresponding glutamatergic subtypes (Figure S1, Supporting Information). To quantitatively subtype identification accuracy, we used cell‐spot mapping matrices constructed by HIDF, Redeconv, Tangram, and GraphST to project true cell subtypes onto spots, and calculated the RMSE between predicted and true proportions. As shown in Figure 2D, HIDF (0.11) demonstrated advantages over Tangram (0.87) and GraphST (1.39). Notably, Redeconv achieved the lowest total RMSE (0.09), within 0.02 of HIDF.

Visualization also showed that neither Tangram nor GraphST accurately identified astrocyte and oligodendrocyte distributions. However, GraphST better recognized neuronal subtype distributions than Tangram (Figure 2E).

To further evaluate the performance of HIDF on simulated datasets, we performed a paired t‐test based on results from seven datasets. The results demonstrated that HIDF has a statistically significant advantage over existing methods (Figure S2, Supporting Information).

We also evaluated the running times of all methods on the simulated datasets (Table S7, Supporting Information). The running time of HIDF gradually increased with the rise in gene expression or pots number. Nevertheless, it remained faster than both Cell2location and Redeconv, which performed well on the simulated data. Notably, Redeconv required an excessively long runtime, over two days on the MERFISH20 dataset. Therefore, we followed the Redeconv official manual. Subsequently, we used the cell type‐level Redeconv deconvolution settings. Redeconv was excluded from the subsequent runtime comparison on the real dataset due to this non‐standard configuration.

These results demonstrate that HIDF's hierarchical architecture enables accurate cell‐type deconvolution. Moreover, benefiting from its hierarchical design, HIDF effectively identified the heterogeneity of cell subtypes within simulated ST data.

### 2.3 HIDF Accurately Identifies Cortical and Hippocampal Architectures in Mouse Anterior‐Posterior Brain and Reveals Spatial Heterogeneity of Astrocyte Subtypes

**Figure 3:**
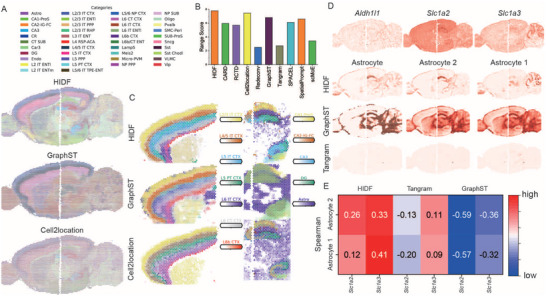
Analysis of mouse anterior and posterior dataset. A) Scatter pie plots show the deconvolution results of the top three methods (HIDF, GraphST, Cell2location) based on comprehensive ranking scores in the mouse brain dataset. B) Bar plot displays the comprehensive ranking scores for all compared methods. C) Spatial distribution patterns of cellular composition of the mouse cerebral cortex and hippocampal dentate gyrus regions. D) Spatial abundance distributions estimated by HIDF are presented for: the overall astrocyte population, its two subtypes, and three astrocyte marker genes. E) Heatmap shows the Spearman's rank correlation coefficients between the spatial expression of marker genes Slc1a2/Slc1a3 and the distributions of two astrocyte subtypes estimated by HIDF, Tangram, and GraphST.

We applied HIDF and mainstream methods to a 10x Visium mouse anterior‐posterior brain ST dataset, covering the cerebral cortex and hippocampal dentate gyrus (DG), to validate HIDF's performance on real biological tissues. As shown in **Figure** **3**A and Figures S3 and S4 (Supporting Information), HIDF clearly identified the anatomical structural features of mouse brain.

We systematically evaluated HIDF and other methods using two metrics: Spearman's ρ (correlating cell‐type abundance with marker gene expression) and Moran's I (measuring spatial autocorrelation of abundance distributions). We specifically calculated these metrics for cortex neuronal subtypes L2‐L6 and hippocampal cell types, including DG, CA1, CA2, and CA3. Cell type abundance heatmaps for all methods (Figures S3 and S4, Supporting Information) and the expression heatmap of cell‐type marker genes (Figure S5, Supporting Information) are provided in the Supporting Information; specific values for the Spearman's ρ and Moran's I are given in Tables S7 and S8 (Supporting Information). To further comprehensively compare the performance of all methods across multiple metrics, we calculated a composite rank score of each method across all metrics (details in Experimental Section).

The comparative analysis demonstrates HIDF's overall superiority, achieving the best composite rank score(Figure 3B). Although Cell2location ranked second overall, it over‐smoothed the data and failed to distinguish hippocampal subfields, such as misplacing CA3 in the DG (Figure 3C). GraphST exhibited strong spatial autocorrelation, with a statistically significant higher Moran's I than HIDF (*p* < 0.01). However, GraphST's Spearman correlation was significantly lower than HIDF's (*p* < 0.01), and it produced a distorted hippocampal structure (Figure 3C). Although CARD achieved the highest average Spearman's ρ, its poor spatial autocorrelation indicated boundary blurring (average Moran's I = 0.63 vs 0.76, *p* < 0.01, Figure S4 and Tables S8 and S9, Supporting Information). RCTD captured macrostructure but had lower Moran's I than HIDF (with *p* < 0.001, Table S8, Supporting Information). Redeconv failed to capture any anatomical features of the mouse brain, such as the hippocampal region. SPACEL and SpatialPrompt generated clear layer structure of cortex, but HIDF outperformed them in marker gene correlation (e.g., hippocampal DG regional average Spearman's ρ = 0.24 vs SpatialPrompt's 0.16, Table S9, Supporting Information). scMoE performed poor on this dataset, possibly due to its failure to incorporate spatial information and not considering for batch effects. Additionally, SpatialPrompt showed lower correlation with astrocyte marker gene expression than HIDF (Spearman's ρ = 0.08 vs. 0.22).

Notably, due to HIDF's unique hierarchical deconvolution architecture, HIDF showed a unique capability to resolve the spatial heterogeneity of cell subtypes. Within the mouse anterior‐posterior dataset, HIDF successfully identified two astrocyte subtypes and revealed their distinct spatial distributions. Subtype 1 was primarily enriched in the hippocampus and adjacent structures, while subtype 2 was specifically enriched in the cerebellar cortex region (Figure 3D). This distribution difference may reflect the region‐specific function of astrocytes in different microenvironments. To validate the biological relevance of subtype identification, we analyzed the regional expression patterns of known astrocyte marker genes. *Slc1a2, RN196* is a typical marker for astrocytes in the brain, especially in synapse‐dense regions like the hippocampus and cortex. While *Slc1a3, RN197* is a specific marker for Bergmann glia in the cerebellar cortex. Spearman's ρ analysis revealed contrasting marker associations: Slc1a2 correlated more with subtype 2, while Slc1a3 correlated more with subtype 1 (Figure 3D, E).

To further validate the reliability of the cell subtypes identified by HIDF, we performed GO enrichment analysis on the top 100 key genes corresponding to these two astrocyte subtypes (Details in “Sensitivity analysis of HIDF parameters” in Supporting Information). As shown in Figure S7 (Supporting Information), astrocyte subtype 1 are significantly enriched in pathways such as “Regulation Of Neuron Projection Development” and “Neuron Projection”. While astrocyte subtype 2 enriched in pathways such as “Regulation Ion Homeostasis” and “Intracellular Potassium Ion Homeostasis” (Figure S8, Supporting Information). These functional enrichments show clear region‐specificity. Subtype 1's role in neuron projection development aligns with Bergmann glia's known function in guiding neuronal migration in the cerebellum.^[^ ^27^, ^28^ ^]^ In contrast, subtype 2's enrichment in ion homeostasis pathways is crucial for the microenvironments of the cortex and hippocampus. Thus, HIDF effectively distinguishes brain region‐specific functional specialization, providing reliable biological interpretation of spatial transcriptomic data. These results, consistent with biological knowledge, validate HIDF's subtype identification and reveal a structural basis for functionally distinct astrocyte subtypes.

To highlight HIDF's advantage in subtype resolution, we compared its performance in identifying astrocyte subtypes with Tangram and GraphST. We mapped the subtypes onto the spatial transcriptomic space via a mapping matrix trained in Tangram and GraphST. Figure 3D,E revealed that neither Tangram nor GraphST can capture the spatial distribution differences of the subtypes; their dot plots showed highly mixed or random patterns. Furthermore, their Spearman's ρ between the subtypes and the markers *Slc1a2*/*Slc1a3* did not show positive correlations (Figure 3E).

We also compared the running times of all methods on the mouse anterior‐posterior brain dataset (Table S7, Supporting Information). It should be noted that neither GraphST nor Tangram could be executed under the 24GB GPU memory constraint. Thus, they were run on CPU and their running times were not included in the comparison. Due to the need for multiple iterations and the construction of a large mapping matrix (116921 × 6050), HIDF exhibited the second‐longest running time on this dataset. To address this limitation of HIDF on large‐scale data, we propose three optimization strategies in the Supporting Information.

In summary, our evaluation on mouse anterior‐posterior brain ST data shows that HIDF outperforms existing methods in both spatial autocorrelation and marker gene correlation. Crucially, its hierarchical architecture uniquely enables it to resolve spatial heterogeneity at the subtype level. The reliability of these results was confirmed using biological markers. These findings indicated that HIDF provided a powerful and robust solution for high‐precision deconvolution of low‐resolution spatial transcriptomic data, enabling the detailed mapping of cell types and their subtype distributions.

### 2.4 HIDF Achieves Precise Deconvolution in Human Breast Cancer and Reveals Tumor‐Region‐Specific Distribution of Luminal Subtypes

Human breast cancer tissue provided an ideal environment for evaluating cell type deconvolution algorithms in highly heterogeneous, complex tissues. This tissue exhibits significant biological heterogeneity, encompassing diverse molecular subtypes, regional variations in tumor cell distribution, and a complex tumor microenvironment. Given that this tissue is inherently a mixture of multiple cell types (e.g., tumor cells, immune cells, fibroblasts), its low‐resolution ST data inevitably contains overlapping cellular signals. Therefore, we applied HIDF and other deconvolution methods to 10x Visium human breast cancer datasets to quantitatively resolve cell‐type proportions and molecular features, systematically evaluating their performance in addressing these complex challenges.

**Figure 4:**
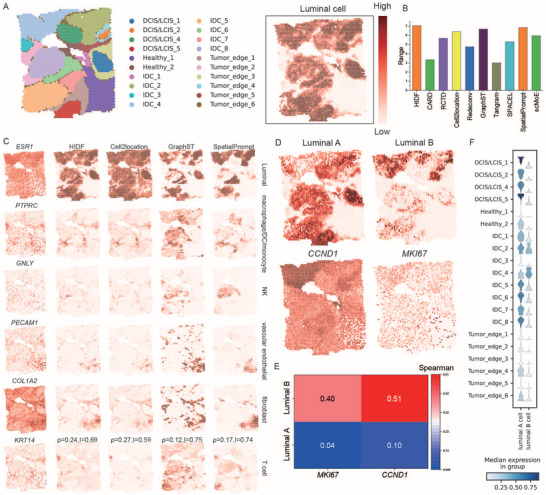
Analysis of the human breast cancer datasets. A) Manually annotated spatial domains and the spatial abundance distribution of luminal cells estimated by HIDF. B) Bar plot displays the comprehensive ranking scores for all compared methods. C) Spatial abundance distributions of selected cell types estimated by the top four methods (HIDF, Cell2location, GraphST, SpatialPrompt), and the spatial expression patterns of their corresponding marker genes. D) Spatial distributions of luminal A and luminal B subtypes estimated by HIDF, and the spatial expression of a luminal B‐specific marker gene. E) Heatmap shows the Spearman's rank correlation coefficients between the spatial distributions of luminal A/B subtypes and the luminal B‐specific marker gene. F) Stacked violin plots show the abundance differences of the two luminal subtypes across different spatial domains.

Spatial abundance heatmaps for luminal cell types showed that HIDF effectively identified the relatively higher expression of luminal cells in the tumor core and invasive front regions (**Figure** **4**A). We calculated the Spearman's ρ and Moran's I again to compare with other methods. Cell type abundance heatmaps for all methods (Figure S9, Supporting Information) and the expression heatmap of cell‐type marker genes (Figure S10, Supporting Information) are provided in the Supporting Information; specific values for the Spearman's ρ and Moran's I are given in Tables S10 and S11 (Supporting Information). As shown in Figure 4B, HIDF achieved the best composite rank score and exhibited the second‐best average Spearman's ρ and the third‐best average Moran's I (Tables S10 and S11, Supporting Information).

In contrast, although Cell2location achieved the highest average Spearman's ρ, its spatial autocorrelation was significantly lower than of HIDF (*p* < 0.001, Figure S11, Supporting Information). For all cell types except pDC, HIDF performed higher Moran's I than Cell2location (Table S10, Supporting Information). Moreover, in identifying the distribution of luminal cells, Cell2location overestimated abundance of luminal cells in healthy regions (Figure 4C). scMoE achieved a higher mean Moran's I, but the difference from HIDF was not statistically significant (*p* = 0.13). In contrast, HIDF significantly outperformed scMoE in Spearman correlation coefficient (*p* < 0.001). Redeconv significantly underperformed HIDF on both metrics (Moran's I, *p* < 0.05; Spearman's ρ, *p* < 0.001) GraphST achieved the highest average Moran's I (0.71). However, deconvolution accuracy was insufficient, evidenced a significantly lower Spearman's ρ compared to HIDF (*p* < 0.01, Figure S11 and Table S11, Supporting Information). For example, as shown in Figure 4C, GraphST exhibited deviation when inferring the spatial distribution of macrophage/DC/monocyte cells. Although GraphST showed high spatial autocorrelation for these cells, the inferred abundance distribution correlated poorly with cell‐type‐specific marker expression. For example, its average Spearman's ρ for markers like *PTPRC* and *ITGAM* was only 0.04, much lower than HIDF's 0.26 (Table S11, Supporting Information). Similarly, spatialPrompt achieved higher Moran's I than HIDF (*p* < 0.05, Figure S11 and Table S9, Supporting Information), but exhibited inadequate deconvolution precision. Its average Spearman's ρ was 0.22, significantly lower than HIDF's (*p* < 0.001; Figure S11 and Table S10, Supporting Information). As shown in Figure 4C, SpatialPrompt's inferred abundances for NK cells, fibroblasts, and myoepithelial cells showed clear inconsistencies with their corresponding marker expression patterns.

Crucially, by leveraging its hierarchical model architecture, HIDF successfully identified two biologically meaningful subtypes within luminal cells: luminal A and luminal B. This identification was supported by two key pieces of evidence: a hierarchical classification tree from the reference data, and differentially expressed features between subclusters—such as MKI67^[^ ^29^, ^30^ ^]^ and CCND1, the latter being a specific marker for luminal B. Deconvolution analysis revealed that these two subtypes exhibited distinct spatial distribution patterns (Figure 4C), and their distributions were consistent with their respective molecular characteristics.

As shown in Figure 4D, we observed that luminal A was the predominant luminal subtype within the DCIS region. Luminal B was more enriched in the IDC1 and IDC2 subregions of invasive ductal carcinoma (IDC) than in other IDC subregions. The spatial distribution of luminal B cells strongly agreed with the expression patterns of its specific markers, CCND1^[^ ^31^ ^]^ and MKI67 (Figure 4C, D). To validate the accuracy of subtype identification, we calculated the Spearman correlation coefficients between luminal A/B cell abundance and the spatial expression of luminal B‐specific markers. Figure 4E revealed moderate correlations between luminal B cells and both markers (Spearman's ρ = 0.40 and 0.51). In contrast, luminal A cells showed weak correlations (Spearman's ρ = 0.04 and 0.10). We performed HIDF parameter sensitivity and GO enrichment analyses on the luminal A and B subtypes, following the same strategy used for the mouse brain dataset. Figure S12 shows significant enrichment of the “Response To Estrogen” pathway in luminal A, which aligns with its classical definition as an estrogen receptor‐positive subtype. In addition, pathways related to “Negative Regulation Of Cell Motility” and “Regulation Of Cell Proliferation” were also significantly enriched in this subtype. In contrast, as shown in Figure S13 (Supporting Information), luminal B exhibited significant enrichment in core biological processes such as “Mitotic Spindle Checkpoint Signaling” and “Mitotic Cell Cycle Phase Transition,” indicating a highly active proliferative state consistent with its known clinical aggressiveness. These enrichment patterns align with the known clinical behavior of luminal A (less aggressive) versus luminal B (more aggressive), supporting HIDF's effectiveness in identifying clinically relevant subtypes.^[^ ^32^, ^33^ ^]^

These results strongly supported the biological reliability of the subtypes identified by HIDF and the accuracy of their spatial distribution patterns.

On the human breast cancer datasets, HIDF demonstrated intermediate runtime among all methods (Table S7, Supporting Information). Notably, when compared to other cell‐level mapping method like Tangram, HIDF required only half the running time.

In conclusion, this study validates HIDF's superior performance in complex human breast cancer tissue. Leveraging its unique hierarchical architecture, HIDF achieved multi‐level resolution of the tumor microenvironment's cellular composition. It effectively identified the differential spatial distribution of luminal cell subtypes, providing a powerful analytical tool for precision oncology research.

### 2.5 HIDF Effectively Reconstructs Spatiotemporal Differentiation Trajectories of T Cells in Mouse Thymus and Resolves Myeloid Subtypes Heterogeneity

In life science research, resolving the spatiotemporal dynamics of cell differentiation is a core challenge for understanding tissue function. Although scRNA‐seq can reveal cellular state heterogeneity, the lack of spatial information makes it difficult to accurately reconstruct continuous differentiation processes dependent on the microenvironment (e.g., T‐cell development). Advances in spatial transcriptomics offer new solutions to this problem. This study systematically compared the performance of HIDF and existing methods in reconstructing T‐cell spatiotemporal differentiation trajectories using a Stereo‐CITE‐seq mouse thymus spatial transcriptomic dataset exhibiting continuous differentiation features.

Spatial regulation tightly governs T‐cell development in the thymus. The process begins with DN precursors differentiating subcapsularly. These cells then transition to the DP stage (co‐expressing *Cd4*/*Cd8a*) in the cortex, undergoing positive selection. Subsequently, immature cells migrate to the corticomedullary junction before final maturation into SP or Treg lineages in the medulla.^[^ ^34^, ^35^, ^36^ ^]^

**Figure 5:**
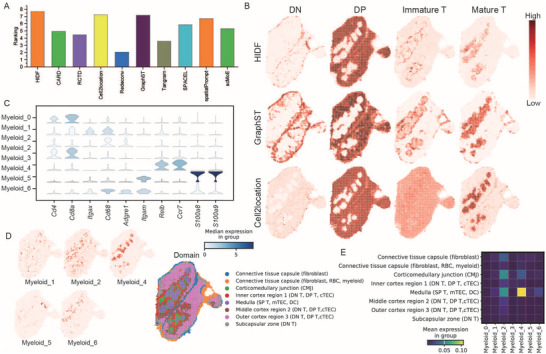
Analysis of the mouse thymus dataset. A) Bar plot showing the comprehensive ranking scores for all compared methods. B) Spatial abundance distribution of T cells at different developmental stages, estimated by the top three ranked methods (HIDF, Cell2location, GraphST). C) Stacked violin plots showing expression levels of marker genes for myeloid cell subtypes, estimated by HIDF. D) Spatial distribution of five myeloid cell subtypes estimated by HIDF. E) Heatmap displaying the distribution features of five myeloid cell subtypes across different spatial domains in the mouse thymus.

To systematically evaluate method performance, we calculated the Spearman's ρ and the Moran's I. As shown in **Figure** **5**A and Tables S12 and S13 (Supporting Information), HIDF achieved the best composite rank score and the second‐best average Spearman's ρ, demonstrating its superior overall performance. HIDF accurately reconstructed T‐cell spatial dynamics(Figure 5B; Figure S14, Supporting Information): 1) DN cells enriched in the subcapsular region (developmental initiation site). 2) DP cells localized to the cortical Cd4/Cd8a co‐expression domain. 3) Immature cells distributed at the corticomedullary junction (matching migration patterns). 4) SP cells specifically aggregated in the central medulla. This spatial architecture fully recapitulated known T‐cell developmental trajectories.

Point biserial correlation analysis further revealed strong associations with the medullary region for both a negative selection subtype (Neg.Sel.2; ρ = 0.52) and Treg cells (ρ = 0.72, *p* < 0.001 for both; Figure S15, Supporting Information). Neg.Sel.2 exhibiting a spatial distribution pattern surrounding Treg cells (Figure S14, Supporting Information), supporting the biological mechanism where negative selection drives Treg maturation.

Comparative methods showed limitations. CARD was insufficient in resolving the spatial correlation of DN cell enrichment in the subcapsular region (point biserial correlation = 0.26 vs. HIDF 0.40, Figure S7, Supporting Information).

Cell2location exhibited bias in localizing DN cells (point biserial correlation = 0.20). It also incorrectly identified DP cells in the subcapsular region, evidenced by a lower average correlation with DP markers (ρ = 0.26) compared to HIDF (ρ = 0.31; Figure 5B; Table S13, Supporting Information). Redeconv only accurately identified Treg cells in the medulla, but failed to detect T cells at other developmental stages. Tangram lacked spatial specificity (lowest Moran's I) and failed to capture T‐cell enrichment in the medulla (Figure S14, Supporting Information). RCTD and SPACEL exhibited significantly weaker spatial autocorrelation than HIDF, with average Moran's I values of 0.33 (*p* < 0.001) and 0.48 (*p* < 0.05), respectively (Figure S15 and Table S12, Supporting Information). GraphST had the lowest expression consistency, such as incorrectly identifying mature T cells in the capsule region (Figure 5B; Table S13, Supporting Information). SpatialPrompt exhibited higher Moran's I than HIDF, but this difference was not statistically significant (*p* > 0.05, Figure S15, Supporting Information). SpatialPrompt also achieved the second‐lowest Spearman's ρ, a value significantly lower than HIDF's (*p* < 0.01; Figure S15, Supporting Information). This limitation is exemplified by its failure to identify the specific distribution of myeloid cells in the medulla (Figure S14 and Tables S12 and S13, Supporting Information). scMoE had lower Moran's I and Spearman values than HIDF. It also failed to resolve the spatial distribution of Immature T and Mature T cells in the mouse thymus.

Notably, HIDF overcame the limitations of existing methods in resolving myeloid cell heterogeneity through its hierarchical deconvolution architecture. As shown in Figure 5C, HIDF identified the coarse‐grained Myeloid annotation into seven subtypes. Clusters 0 and 3, with high expression of DP cell marker *Cd4*/*Cd8a, RN209*, were redefined as DP cells. It was supported by GO enrichment showing significant enrichment for T cell‐related pathways (e.g., “T cell activation” and “T cell differentiation”, see in Figure S16, Supporting Information), indicating the original annotation required revision. Cluster 2, characterized by high *Cd68*/*Adgre1* expression, was identified as macrophages with these macrophage markers.^[^ ^37^ ^]^ GO enrichment results of its “Phagocytosis” and “Apoptotic cell clearance” confirmed this (Figure S17, Supporting Information). Figure 5D,E showed its spatial enrichment in the corticomedullary junction (CMJ) and medulla. Cluster 1 (high *Itgax* expression) and Cluster 6 (high *Itgax*/*Itgam* expression) localized to the medulla. Cluster 4 was enriched in the inner medulla and highly expressed two migration‐related genes: *Relb* and *Ccr7* (Figure 5C–E). The spatial abundance of Cluster 4 weakly correlated with *Relb* (Spearman's ρ = 0.11, *p* < 0.001) and *Ccr7* (Spearman's ρ = 0.14, *p* < 0.001). GO enrichment analysis of these potential DC subsets revealed pathways closely related to their functions (Figure S18, Supporting Information): Cluster 1 was enriched in “Antigen Processing and Presentation of Peptide Antigen via MHC Class I”,^[^ ^38^ ^]^ Cluster 4 in “Response to type II interferon”,^[^ ^39^ ^]^ and Cluster 6 in “Fc‐gamma receptor signaling pathway”.^[^ ^40^ ^]^ Based on their marker genes (*Itgax*, *Itgam, RN211*) and the pathway enrichment results above, Clusters 1, 4, and 6 were identified as dendritic cells. Cluster 5 was identified as neutrophils due to its high expression of canonical neutrophil markers *Itgam*, *S100a8*, and *S100a9, RN212*. Its enrichment analysis pointed to “Neutrophil Activation” and “Neutrophil Migration” pathways (Figure S19, Supporting Information). Spatial localization analysis indicated it was primarily enriched near the subcapsular vascular region of the thymus (Figure 5C,D).

Regarding the running time on the mouse thymus dataset, HIDF demonstrated advantages in both efficiency and performance, with a running time approximately half that of Tangram and the second‐best method, Cell2location (Table S7, Supporting Information).

In summary, through its hierarchical resolution strategy, HIDF overcame the limitations of existing methods in overlooking the coarse nature of single‐cell annotations. It not only corrected DP cell misannotation within the myeloid population but also revealed spatially specialized functional patterns of macrophage and dendritic cell subpopulations. This study confirmed that HIDF provides an efficient and precise computational framework for resolving cellular heterogeneity and spatial organization rules in complex tissues.

### 2.6 Evaluation of HIDF's Generalization in Cross‐Species Deconvolution

In real‐world applications, distribution shifts often exist between reference single‐cell data and target spatial transcriptomics (ST) data, such as when data come from different donors, tissues, or species. To investigate the robustness of HIDF under such OOD settings, we performed cross‐species deconvolution using a mouse brain cortex dataset as the reference on 10x Visium human DLPFC dataset with clear layered structures.

**Figure 6:**
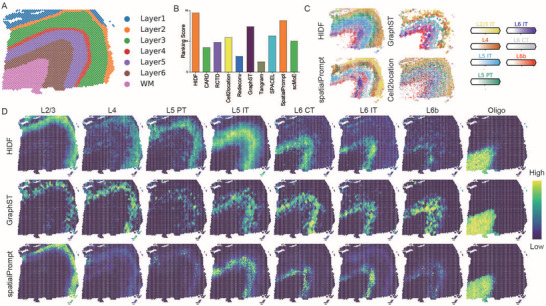
A) Spatial domains of the human DLPFC dataset. B) Bar plot showing the comprehensive ranking scores for all compared methods. C,D) Heatmaps of neuronal subtype distribution for HIDF, spatialPrompt, GraphST, and Cell2location.

To quantitatively evaluate the methods, we computed both the Moran's I and the point‐biserial correlation with respect to cortical layers, combining them into a comprehensive ranking score. As shown in **Figure** **6**A,B, HIDF achieved the highest ranking score and significantly outperformed most existing methods (except GraphST) in both metrics (*p* < 0.05, Figure S20 and Tables S14 and S15, Supporting Information). However, although GraphST did not differ significantly from HIDF in Moran's I and point‐biserial, its deconvolution results exhibited visually less spatial continuity (Figure 6C,D; Figure S21, Supporting Information).

On the DLPFC dataset, most methods show generally poor deconvolution performance. This is likely due to the dataset's strong spatial autocorrelation and substantial distributional differences from the reference single‐cell data. Methods that fail to effectively utilize spatial information or correct for distribution shifts may struggle to achieve accurate deconvolution. For scMoE and Redeconv, their inability to explicitly model spatial structure or handle batch effects may explain its suboptimal performance on this dataset. Regarding running time on human DLPFC dataset, HIDF achieved a middle‐tier running time, while being notably faster than several GPU‐compatible methods, including Tangram, Cell2location, and SPACEL (Table S7, Supporting Information).

These results demonstrate that HIDF can accurately recover structural patterns and produce biologically interpretable outputs even in challenging cross‐species settings, confirming the robustness of our method.

### 2.7 Evaluating HIDF on Next‐Generation ST Platforms

HIDF is primarily designed for platforms with complex cell type composition but relatively low spatial resolution. However, next‐generation spatial transcriptomics technologies such as Xenium and Visium HD now provide significantly higher resolution, reaching single‐cell or even subcellular levels. To evaluate HIDF's performance on such high‐resolution data, we conducted a deconvolution experiment using a mouse brain dataset generated by Xenium (36,602 cells, 248 genes). A mouse whole‐brain single‐cell transcriptomic dataset (116,921 cells) was used as reference.

It should be noted that when both spot and cell numbers are extremely large, although HIDF is well‐optimized, it could not run directly under the 24GB GPU memory constraint. To address this hardware limitation, we proposed three feasible solutions in the supplementary text. In this experiment, we applied the first strategy: downsampling the reference cell set. Furthermore, for high‐resolution data like Xenium, HIDF's fundamental assumption that one spot contains multiple cells may no longer strictly hold. This makes the experiment particularly challenging for HIDF.

Nevertheless, HIDF still demonstrated competitive deconvolution performance on the Xenium data (Figures S22–S25 and Table S16, Supporting Information). Specifically, it clearly reconstructed the layered structure of the mouse cortex and accurately identified cells in the hippocampal dentate gyrus (DG) and its subregions (CA1, CA2, CA3). A key advantage was observed in CA2 identification, methods including CARD, Cell2location, GraphST, and Tangram failed to detect CA2's specific distribution. In contrast, HIDF, RCTD, Redeconv, SPACEL, SpatialPrompt, and scMoE successfully identified CA2's characteristic localization between CA1 and CA3.

We computed Spearman correlation coefficients for selected cell types and their corresponding markers. Figure S25 (Supporting Information) shows that HIDF performs at a moderate level, with no significant difference compared to most other methods (*p* > 0.05). Notably, scMoE showed average performance on previous low‐resolution datasets but performed notably better on the Xenium dataset. This may be because scMoE was originally designed for single‐cell transcriptomic data, making it more suitable for high‐resolution spatial transcriptomics datasets such as Xenium.

Since Xenium data approach single‐cell resolution, the predicted cell type composition for each spot should ideally be highly certain, as reflected by low entropy. Therefore, We compared the average entropy of the predictions across methods to assess their certainty. HIDF achieved the second‐lowest average entropy, performing significantly better than all other compared methods (Figure S25, Supporting Information). Redeconv exhibited the lowest information entropy, significant lower than HIDF (*p* < 0.001, Figure S25, Supporting Information). However, HIDF demonstrated a significantly higher Spearman correlation than Redeconv (*p* < 0.01, Figure S25, Supporting Information). Thus, although Redeconv has slightly higher certainty, HIDF shows overall better performance. On Xenium dataset, HIDF showed the slowest performance among all methods. This may be attributed to the relatively large number of spots, which appears to significantly impact the computational efficiency of HIDF. We have discussed this limitation and potential optimizations in the supplementary text.

In summary, although HIDF was not specifically designed for ultra‐high‐resolution technologies like Xenium, it still delivered competitive cell deconvolution results on such data, demonstrating good robustness and generalization potential.

### 2.8 Ablation Experiment

To quantify the contributions of individual modules in HIDF, we constructed three ablation variants on simulated seqFISH and MERFISH datasets.

i) HIDF‐w/o crc denotes the model with the cross‐level regularization constraint removed.

ii) HIDF‐w/o src denotes the model with the spatial regularization constraint removed.

iii) HIDF‐w/o h denotes the model without hierarchical deconvolution, directly constructing and optimizing the cell‐spot mapping matrix.

**Figure 7:**
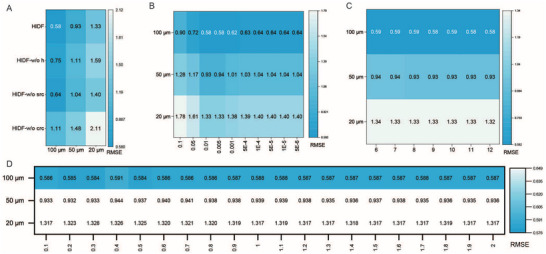
Results of ablation experiments and parameter analysis on MERFISH dataset. A) Ablation analysis of different module. B) Effect of the spatial regularization parameter λ on model performance. C) Effect of the neighbor count *k* on model performance. D) Effect of the Leiden resolution parameter on model performance.

As shown in **Figure** **7**A, removing either cross‐level constraints or the iterative deconvolution process degraded model performance. On the MERFISH dataset, disabling cross‐level constraints reduced HIDF's accuracy by 0.53, 0.55, and 0.78 across evaluation metrics. In the seqFISH dataset (Figure S26, Supporting Information), spatial regularization negatively impacted performance (average RMSE increase 0.09), likely due to low spatial autocorrelation weakening neighborhood constraints. Nevertheless, the full HIDF maintained best performance, reducing RMSE by 11.1%, 16.9%, and 17.1% compared to the second‐best method RCTD. Conversely, in the MERFISH dataset with high spatial autocorrelation, spatial regularization reduced RMSE by 0.06, 0.11, and 0.07 across resolutions. These results demonstrated that hierarchical iterative architecture is essential for HIDF. Its absence reduced performance on both datasets, validating the recursive decomposition strategy's importance for precise spatial cell‐type resolution. Among dual regularization constraints, cross‐level constraints significantly influenced performance. Their removal caused the largest RMSE increase (0.78) in MERFISH. Spatial regularization substantially improved results in datasets with high autocorrelation (e.g., MERFISH). HIDF consistently outperformed existing methods, even under adverse conditions like low autocorrelation (seqFISH). In summary, this ablation study confirmed the effectiveness and synergistic value of each module in HIDF.

### 2.9 Parameter Analysis

To evaluate the impact of critical parameters on the performance of the HIDF, we systematically quantified the sensitivity of the spatial regularization coefficient λ, neighborhood size *k*, and leiden clustering resolution *r* using simulated MERFISH and seqFISH datasets.

As shown in Figure 6B, the spatial regularization coefficient λ exhibited a clear optimal range in MERFISH datasets. Excessively low values (λ < 0.001) degraded performance due to ineffective integration of neighborhood cellular composition information, while excessively high values (λ > 0.1) induced over‐smoothing effects. c λ = 0.01, HIDF achieved best RMSE on both MERFISH and seqFISH datasets (Figures 7B; Figure S26B, Supporting Information). This value was set as the default for all analyses on mouse brain, human breast cancer, and mouse thymus datasets. The neighborhood number *k* and leiden resolution parameters demonstrated strong robustness (Figure 7C,D; Figure S26C,D, Supporting Information). Within the ranges *k* ∈ [6, 12] and *r* ∈ [0.1, 2.0], model performance fluctuated within about 0.01, indicating HIDF's insensitivity to these two parameters.

## 3 Conclusion

This study addresses the critical limitation of existing deconvolution methods in resolving cellular heterogeneity within low‐resolution spatial transcriptomics data by introducing the Hierarchical Iterative Deconvolution Framework. HIDF pioneers a three‐stage architecture: 1) constructing a multi‐scale cell reference atlas via unsupervised hierarchical clustering of scRNA‐seq data to capture transcriptional heterogeneity; 2) implementing iterative hierarchical optimization that first resolves coarse‐grained cell type distributions, then recursively decomposes subtypes through cluster‐tree‐guided weight splitting; 3) enforcing dual regularization—spatial smoothness via neighborhood constraints and cross‐level consistency to ensure biological plausibility and escape local optima.

We demonstrate the superior performance of HIDF on both simulated and real‐tissue datasets. Analysis of simulated datasets indicate that HIDF accurately resolves cell type composition. Furthermore, leveraging its hierarchical architecture, HIDF successfully identifies hierarchical subtype distributions of glutamatergic neurons. Evaluation on real datasets further confirms HIDF's effectiveness, it achieves the highest score on comprehensive evaluation metrics, enabling highly efficient cell type deconvolution, and reveals key subtype structures missed by other methods. In mouse anterior–posterior brain tissue, HIDF identifies astrocyte subtypes with markedly distinct spatial distribution patterns. In human breast cancer samples, HIDF uncovers significant differences in the spatial distribution patterns between luminal A and luminal B subtypes. In mouse thymus tissue, HIDF corrects erroneous annotations of myeloid cells and further discovers spatially heterogeneous distribution features among myeloid subtypes, including macrophages and dendritic cells. In DLPFC dataset, HIDF demonstrated robust generalization in cross‐species mapping and achieved superior performance compared to existing methods. In Xenium dataset, HIDF's linear framework and mini‐batch training provide inherent scalability for this dataset. While the current HIDF may face GPU memory constraints with extremely large references, we have outlined clear pathways for future enhancement, including the adoption of sparse matrix operations and strategic cell pruning.

In conclusion, by breaking through the limitations of fixed cell type annotation and effectively leveraging spatial heterogeneity information, HIDF overcomes key constraints of existing methods. It establishes a powerful deconvolution framework that maps transcriptomic hierarchies onto spatial tissue architectures.

## 4 Experimental Section

### Data Preprocessing

First, the common gene set shared between ST and scRNA‐seq data was identified. Based on the scRNA‐seq data, differential expression analysis was performed grouped by cell type (with default thresholds $\left|log_{2}^{FC}\right|>0.5$ and *adj* − *pval* < 0.01) to extract differentially expressed genes (DEGs). Subsequently, based on these DEGs, normalization was conducted using the scanpy^[^ ^41^ ^]^ toolkit. The ST data underwent library size normalization (target sum = 10,000) followed by log transformation, while the scRNA‐seq data received only library size normalization (target sum = 10,000).

### Hierarchical Tree Construction

A hierarchical partitioning strategy was employed based on the scRNA‐seq reference dataset to construct a hierarchical cluster tree. The specific construction workflow was as follows.

First, predefined cell type annotations in the reference dataset served as initial clusters. Next, for each cell type cluster, the leiden^[^ ^42^ ^]^ clustering (resolution parameter = 0.3) was applied recursively to partition it into subclusters based on the similarity of gene expression profiles among cells. The partitioning iteration continued until one of the following conditions was met: the current cluster contained fewer than 20 cells, or Leiden clustering identified only a single cluster within it. The resulting hierarchical tree comprised non‐leaf nodes, representing cell populations at varying granularities (coarser classification units), and leaf nodes, corresponding to individual cells. This structure establishes a multiscale representation framework, ranging from the macro level (cell type) through the meso level (subtype) down to the micro level (single cell).

### Hierarchical Iterative Deconvolution Framework

This framework employs a hierarchical iterative optimization strategy. Its core workflow is as follows.

Starting from all nodes at the current level of the cluster tree, the mean gene expression vector μ for each node's corresponding cell cluster was calculated. This serves as the reference cell prototype for spatial deconvolution, with the formula as follows:

(1) $$\mu_{n}=\frac{1}{\left|C_{n}\right|}\sum_{c\in C_{n}}x_{c}$$

where *n* represents tree nodes, *C*_*n*_ denotes the set of cells associated with node, *x*_*c*_ is the gene expression vector of cell *c*, and |*C*_*n*_| is the number of cells in the set.

The constrained spatial mapping model was adapted from RCTD.^[^ ^15^ ^]^ For each spatial spot *s*, the gene expression reconstruction formula is as follows.

(2) $$x_{si}^{\prime }=\sum_{p}^{P}\log\left(1+w_{sp}{\cdot \mu }_{pi}\right)+\gamma_{i}+\beta_{s}$$

where *w*_*sp*_ is subjected to the constraint $\sum_{p}^{p}w_{sp}=1$. Here, *x*_*si*_ is the reconstructed expression value of gene *i* in spot *s*, *w*_*sp*_ represents the proportion weight of prototype *p* in *s*, μ_*pi*_ is the expression value of gene *i* in prototype *p*, γ_*i*_ represents gene‐specific batch offset, and β_*s*_ denotes spot‐specific technical bias. The reconstruction loss utilizes cosine similarity loss, defined as follows.

(3) $$L_{cosine}=\frac{1}{S}\sum_{s=0}^{S}\left(1-\frac{x_{s}\cdot x_{s}^{\prime }}{|x_{s}\left|\right|x_{s}^{\prime }|}\right)$$

where *S* is the total number of spots, and *x*_*s*_ and $x_{s}^{\prime }$ are the original and reconstructed expression vectors for spot *s*, respectively.

Upon convergence at the current hierarchical level, the process advances to child levels within the cluster tree. For new child nodes, child level cell prototypes were generated using Formula (1). The child level inherits the mapping weights from its parent level. After a parent cluster *p* splits into *m* child clusters *p*_1_, *p*_2_, …, *p*_*m*_, the weights for the child clusters are initialized as:

(4) $$w_{sp_{k}}^{\mathrm{child}}=\frac{w_{sp}^{\mathrm{parent}}}{m},\forall k\;\in \left\{1,\;2,\;\text{…},\;m\right\}$$

After updating the reference cell prototypes and the mapping matrix, model training resumed to optimize the parameters *w*_*sp*_, γ_*i*_, and β_*s*_. The above steps are repeated recursively until the leaf node (single‐cell) level is reached. This achieves progressively increasing resolution with each hierarchical refinement.

### Cell Type Abundance Estimation

Following model convergence, the proportion of cell type *c* in spot *s* is calculated by aggregating the weights *w*_*sp*_ associated with its reference prototypes:

(5) $$Abundance_{sc}=\sum_{p\in P_{c}}w_{sp}$$

where $P_{c}$ is the set of prototypes corresponding to cell type *c*, and *Abundance*_*sc*_ represents the proportion of cell type *c* in spot *s*.

### Dual Regularization Constraints

To ensure consistency in cell type abundance estimated between parent and child levels, a cross‐level regularization constraint was implemented. An inter‐level mean squared error (MSE) loss is defined as:

(6) $$Abundance_{sc}^{parent}=\sum_{p\in P_{c}^{parent}}w_{sp},\;\;Abundance_{sc}^{child}=\sum_{p\in P_{c}^{child}}w_{sp}$$

(7) $$L_{consistency}=\frac{1}{S}\sum_{s}\sum_{c=1}^{C}{\left(Abundance_{sc}^{parent}-Abundance_{sc}^{child}\right)}^{2}$$

where $Abundance_{sc}^{parent}$ and $Abundance_{sc}^{child}$ represent the abundance of cell type *c* in spot *s* at the parent and child levels, respectively, $P_{c}^{parent}$ and $P_{c}^{child}$ denote the sets of reference prototypes for cell type *c* at the corresponding levels, and *C* is the total number of cell types.

To enable mini‐batch training while preserving spatial continuity, a dynamic memory bank was designed. A continuously updated memory bank *M* ∈ *R* ^*SC*^ stores historical cell type abundances for all spatial spots, where *M*_*sc*_ records the most recent abundance of cell type *c* in spot *s*.

After each training batch, the memory bank was updated using the newly computed abundances.

(8) $$M_{sc}^{new}\leftarrow Abundance_{sc}$$

Spatial regularization was introduced to ensure consistency in the composition of adjacent spot cell types. Based on a k‐nearest neighbor graph (k is dataset‐specific), the neighborhood *N*(*s*) was defined for each spot ss. The constraint enforces consistency between current batch abundances and neighborhood abundances stored in the memory bank.

(9) $$L_{spatial}=\frac{1}{S}\sum_{s}\sum_{s^{\prime }\in N\left(s\right)}\sum_{c=1}^{C}{\left(Abundance_{sc}-M_{s^{\prime }c}\right)}^{2}$$

### Loss Function And Training Strategy

The total loss function of HIDF comprises three components: the reconstruction loss, the cross‐level constraint loss, and the spatial regularization loss. The reconstruction loss $L_{cosine}$ measured the difference in cosine similarity between the reconstructed ST data and the original ST data. The cross‐level constraint loss $L_{consistency}$ enforces consistency in cell type abundance across different levels. This constraint loss was excluded during optimization at the first level. The spatial regularization loss $L_{spatial}$ ensured the spatial continuity of cell abundance distribution.

HIDF was trained using a hierarchical iterative strategy. First, starting at the cell type level, reference data was constructed according to Formula 1. HIDF was then trained using reconstruction loss $L_{cosine}$ and spatial regularization loss $L_{spatial}$. Once convergence was achieved at this level, reference data for the sub‐level was constructed based on the established hierarchical tree, again using Formula 1. Subsequently, the mapping matrix parameters trained at the parent level were used to initialize the sub‐level mapping matrix. Combining Formula 4, the child level model was trained with $L_{cosine}$ and $L_{spatial}$. Additionally, the consistency loss $L_{consistency}$ was introduced to enforce agreement in the abundance of corresponding cell types between parent and child levels (abundance calculated as per Formula 5). This process iterated downward, refining HIDF layer by layer. Training terminated once all reference data corresponded to the single‐cell level.

The overall training loss for HIDF is defined by the following equation:

(10) $$L_{total}=L_{cosine}+L_{consistency}+\lambda \cdot L_{spatial}$$

where the hyperparameter λ controls the strength of spatial regularization, with a default value of 0.01.

### Evaluation Metrics

On the simulated datasets, since the true composition of cell types was known, the Root Mean Square Error(RMSE) was used to evaluate the discrepancy between the predicted cell type proportions and the ground truth. Its calculation formula is as follows:

(11) $$RMSE=\sqrt{\frac{1}{N\times C}\sum_{i=1}^{N}\sum_{c=1}^{C}{\left({\hat{p}}_{ic}-p_{ic}\right)}^{2}}$$

where *N* is the total number of spots, and *C* it the total number of cell types.

On the real datasets, four metrics was used for evaluation, Spearman's rank correlation coefficient (ρ), Moran's I (*I*), Point‐Biserial Correlation Coefficient (*r*_*pb*_) and Entropy (*H*).

Spearman was used to evaluate the correlation between the predicted abundance of a cell type and the expression of its corresponding marker genes. The calculation formula is:

(12) $$\rho =1-\frac{6\sum_{i=1}^{n}d_{i}^{2}}{n(n^{2}-1)}$$

A value closer to 1 indicates a stronger positive correlation between the predicted cell type abundance and marker gene expression.

Moran's I was used to evaluate the spatial autocorrelation of the predicted cell type abundance. The calculation formula is:

(13) $$I=\frac{n}{\sum_{i=1}^{n}\sum_{j=1}^{n}w_{ij}}\cdot \frac{\sum_{i=1}^{n}\sum_{j=1}^{n}w_{ij}\left(x_{i}-\bar{x}\right)\left(x_{j}-\bar{x}\right)}{\sum_{i=1}^{n}{\left(x_{i}-\bar{x}\right)}^{2}}$$

where *x*_*i*_ is the predicted abundance at spot *I*, $\hat{x}$ is the mean abundance, *w*_*ij*_ is the spatial weight matrix, and *n* is the total number of spots. *I* > 0 indicates positive spatial autocorrelation, *I* < 0 indicates negative spatial autocorrelation, and a larger absolute value indicates stronger spatial clustering.

Point‐Biserial correlation *r*_*pb*_ was used to assess the relationship between cell type abundance distribution and corresponding spatial domains. The calculation formula is:

(14) $$r_{pb}=\frac{{\bar{P}}_{1}-{\bar{P}}_{0}}{s_{p}}\sqrt{\frac{p_{1}p_{0}}{p(p-1)}}$$

where ${\bar{P}}_{1}$ and ${\bar{P}}_{0}$ are the mean cell abundance values for group 1 (inside the spatial domain) and group 0 (outside the spatial domain), respectively. *s*_*p*_ is the standard deviation of the continuous variable for the entire sample. *p*_1_ and *p*_0_ are the numbers of observations in group 1 and group 0, respectively. *p* is the total number of observations. A value of *r*_*pb*_ approaching 1 indicates markedly higher abundance inside the domain; a value approaching ‐1 indicates markedly lower abundance inside.

Information Entropy *H* was used to evaluate the certainty of the model‐predicted cell type abundance. The calculation formula is:

(15) $$H=-\sum_{c=1}^{C}p_{c}\cdot \log_{2}\left(p_{c}\right)$$

where *C* is the total number of cell types. *p*_*c*_ represents the probability of the spot being predicted as cell type *c*. *H* is the calculated information entropy value. A lower entropy value *H* reflects a more confident prediction, while a higher *H* indicates greater uncertainty in the cell type assignment.

To comprehensively assess model performance, the two metrics was integrated to define a Composite Rank Score (CRS). For each metric *k* ∈ {ρ, *I*, *r*_*pb*_, *H*}, all methods being compared were ranked based on their evaluation results, sorted in ascending order. Let *Rank*_*k*_(*m*) denote the rank of method *m* on metric *k* (with the worst performer assigned rank 1). For method *m*, compute the average of its ranks across the two metrics:

(16) $$CRS\left(m\right)=\frac{Rank_{k1}\left(m\right)+Rank_{k2}\left(m\right)}{2}$$

The selection of *k*1 and *k*2 depends on which two evaluation metrics were used in the specific experiment.

### Statics Analysis

To evaluate the significance of differences between methods across datasets, a one‐sided paired t‐test was employed. The results were presented as box plots (box: 25th–75th percentiles; whiskers: 1.5×IQR; horizontal line: mean), with the specific sample size (n) for each analysis provided in the figure legend. Statistical analyses were performed using the Scipy package in Python.

## Conflict of Interest

The authors declare no conflict of interest.

## Supporting information



Supporting Information

## Data Availability

Code of HIDF is available at github. The scRNA‐seq reference dataset for the hierarchical simulated dataset is available at GSE115746. The process of generating the simulated ST data is in our code. The descriptions and download paths of other datasets used in this paper can be found in the Tables S18‐S19 in the supplementary information.
