# Supplementary material for: HIDF: Integrating Tree‐Structured scRNA‐seq Heterogeneity for Hierarchical Deconvolution of Spatial Transcriptomics
Source: Adv Sci (Weinh). 2025 Dec 12;13(12):e14073. doi: 10.1002/advs.202514073 (PMC12948217; doi:10.1002/advs.202514073)
Supplement: Supplementary file 1 — Supporting Information [file ADVS-13-e14073-s001.docx]

**Supplementary Text:**

**Hierarchical Dataset simulation process:**

This study simulated spatial transcriptomic data by constructing a 30×15 two-dimensional spatial grid. The specific process is as follows: First, six fixed positions (2.5, 7.5, 12.5, 17.5, 22.5, 27.5) were preset along the X-axis as the central points for cell type distribution. Based on the Gaussian kernel function, the distance weights from the X-coordinate of each grid point to each central point were calculated, with the kernel width controlled by the parameter sigma. Subsequently, based on these unnormalized weights, sampling was conducted from a pre-annotated single-cell RNA reference dataset (containing astrocytes, oligodendrocyte and four neuron subtypes) to generate the cell composition of each spatial site, with 5 cells sampled per site. Finally, the gene expression values of the sampled cells were aggregated to form a spatial expression matrix, and spatial metadata such as site coordinates and cell type proportion distribution were recorded simultaneously, thus forming a spatial distribution pattern of cell types with Gaussian gradient characteristics along the X-axis.

**Comprehensive Comparison between HIDF and Existing Methods:**

To further illustrate the differences and unique advantages of HIDF compared with existing methods, we elaborate on this from the following four aspects: Model Complexity, Computational Cost, Interpretability, and Input Requirements.

We demonstrate this through the Table S17.

**Model complexity.** To ensure a fair comparison of methods with fundamental technical differences in programming language and runtime environment, we adopt theoretical complexity analysis as a unified criterion. Based on algorithmic principles and computational characteristics, existing methods can be categorized into three classes: (1) Low-complexity methods (e.g., CARD, RCTD) have a complexity of Ω(TM), with model size growing linearly with the number of cell types T and spots M. (2) High-complexity methods (e.g., Tangram, GraphST) have a complexity of Ω(NM), as they require constructing a full cell–spot mapping matrix, leading to high complexity when the number of cells N and spots M is large.
(3) Variable-complexity methods (e.g., SPACEL, spatialPrompt) exhibit complexity that depends on specific implementations such as internal neural networks or KNN search.

In contrast, HIDF achieves a good balance between resolution and efficiency in terms of model complexity. Although it also constructs a fine-grained cell–spot mapping matrix, its core mechanism relies on a linear model with iterative optimization, which is simpler than end-to-end deep learning models. By introducing a mini-batch training strategy, HIDF decomposes the large-scale optimization problem, reducing per-iteration complexity from O(NM) to O(NB). Thus, HIDF maintains high resolution while achieving more controllable complexity than typical deep learning methods, and is classified as a deconvolution method with variable complexity.

**Computational Cost.** We evaluated the computational cost of all methods by measuring their running time across datasets. Note that due to differences in training epochs, workflows, and optimization strategies, theoretical complexity does not fully determine actual running time. In our runtime comparison, we excluded Redeconv for its prohibitive runtime, and GraphST and Tangram due to GPU memory constraints. Methods were categorized into three groups: Low (CARD, GraphST, Tangram, spatialPrompt), Medium (HIDF, RCTD), and High (Cell2location, Redeconv, SPACEL). The running time of HIDF is mainly influenced by three factors: **(1) the number of query points**, as seen in MERFISH datasets where runtime rose from 22 s to 427 s with increasing points, though still more scalable than Redeconv; (**2) the number of shared genes**, as shown in seqFISH datasets where runtime increased modestly from 9 s to 26 s; and **(3) the number of reference cells**, where large reference sets (e.g., mouse brain) led to longer runtimes (up to 3442 s). Despite this, HIDF generally maintained reasonable running times across most datasets, without the extreme delays seen in Redeconv, Cell2location, or SPACEL. Moreover, its runtime increased steadily with data scale, reflecting good scalability and computational stability.

**Interpretability.** Interpretability. We systematically assessed the interpretability of all methods, highlighting HIDF’s unique capability to automatically infer hierarchical cell relationships. Existing approaches were categorized into three groups: pseudo-spot-based, cell type-level mapping, and cell-level mapping methods.

(1) Pseudo-spot-based methods such as spatialPrompt and deep learning-based frameworks like SPACEL and GraphST suffer from limited interpretability due to their black-box nature.

(2) Cell type-level mapping methods like CARD offer constrained interpretability that relies entirely on predefined annotations.

(3) Cell-level mapping methods, including Redeconv and HIDF, provide transparent, fine-grained interpretability by revealing each cell’s contribution to each spot.

Notably, HIDF further distinguishes itself by automatically inferring hierarchical cellular relationships directly from data, without relying on prior knowledge, a feature lacking in other cell-resolution methods such as Tangram and Redeconv.

**Input Requirements.** Most methods, including HIDF and Tangram, require only standard reference and query datasets. A key exception is scMoE, which mandatorily uses a manually defined cell lineage tree as prior knowledge. Although HIDF also leverages hierarchical structures, it automatically infers them, eliminating this manual requirement.

**Experimental Setup：**

To ensure reproducibility and fair comparison, all methods were executed under a fixed random seed. We used paired t-tests to evaluate the statistical significance of performance differences. All methods were executed using the template configurations and default parameters specified in their official manuals. Subsequently, all methods were run on identical hardware configurations, and their execution times were recorded for a fair comparison. Hardware configuration is as follows: CPU, AMD Ryzen 9 5950X; Memory, 128 GB RAM; GPU, NVIDIA RTX 4090D(24 GB memory).

**Sensitivity analysis of HIDF parameters:**

After HIDF is trained, the reconstructed gene expression in spatial transcriptomics (ST) is formulated as follows:

$$x_{si}= \sum_{p}^{P} log(1+w_{sp} \mu_{pi})+\gamma_{i}+\beta_{s} (1)$$

Here, $w_{sp}$ denotes the relational weight between the *s*-th spot and the *p*-th cell. To evaluate the influence of a specific cell type on gene expression, we mask the weights $w_{sp}$ corresponding to that cell type *c* by setting them to zero ,resulting in a new weight matrix $w_{sp}^{c}$. By substituting $w_{sp}^{c}$ into the formula (1), we obtain the maksed spot gene expression $x_{si}^{c}$.

Then we compute the variance of the differences in gene expression before and after masking.

$$d_{i}^{c} = {\sum_{s}^{S} \left( x_{si}^{c}- x_{si} \right)^{2}}$$

This variance $d_{i}^{c}$ reflects the overall impact of that cell type *c* on gene *i*. Finally, $d_{i}^{c}$are ranked in descending order, indicating the relative influence of the cell type on each gene’s expression.

**Optimization Strategies for HIDF on Large-Scale Data：**

HIDF requires the construction of a large mapping matrix, which leads to significant hardware burden when applied to extremely large datasets. This study analyzes the key challenges faced by HIDF and proposes corresponding optimization strategies.

Challenge of Reference Cell Number (N): Through optimization, HIDF reduces the per-iteration complexity to O(NB), where B is an adjustable batch size and N is the number of reference cells. However, when N is very large, the computational load remains substantial. We propose three strategies to address this issue: **(1) Downsampling the reference dataset.** Downsampling the single-cell reference dataset can reduce the number of reference cells N. This approach effectively alleviates computational load but may result in the loss of critical cell information, thereby affecting deconvolution accuracy. **(2) Early stopping of iterations.** HIDF only constructs the full N×B mapping matrix when iterations reach all leaf nodes. Therefore, the training process can be stopped early before full convergence to obtain intermediate outputs. This strategy still provides cell-type abundance information, but it cannot yield single-cell resolution deconvolution results. **(3) Pruning.** During the iterative optimization, some cells exhibit very low weights with all spots. Such cells can be dynamically removed during the process to reduce computational complexity. However, the pruning threshold must be set carefully to avoid removing cells that may become important in later iterations.

Challenge of Mapping Matrix Storage (O(NM)): HIDF needs to store the entire mapping matrix in memory, which imposes considerable hardware resource demands. We observe that each spot typically has significant associations with only a small number of reference cells. Therefore, cell–spot combinations with low weights can be masked, and the mapping matrix can be stored in a sparse format. Using sparse representation can significantly reduce memory and GPU usage and improve the efficiency of subsequent computations.


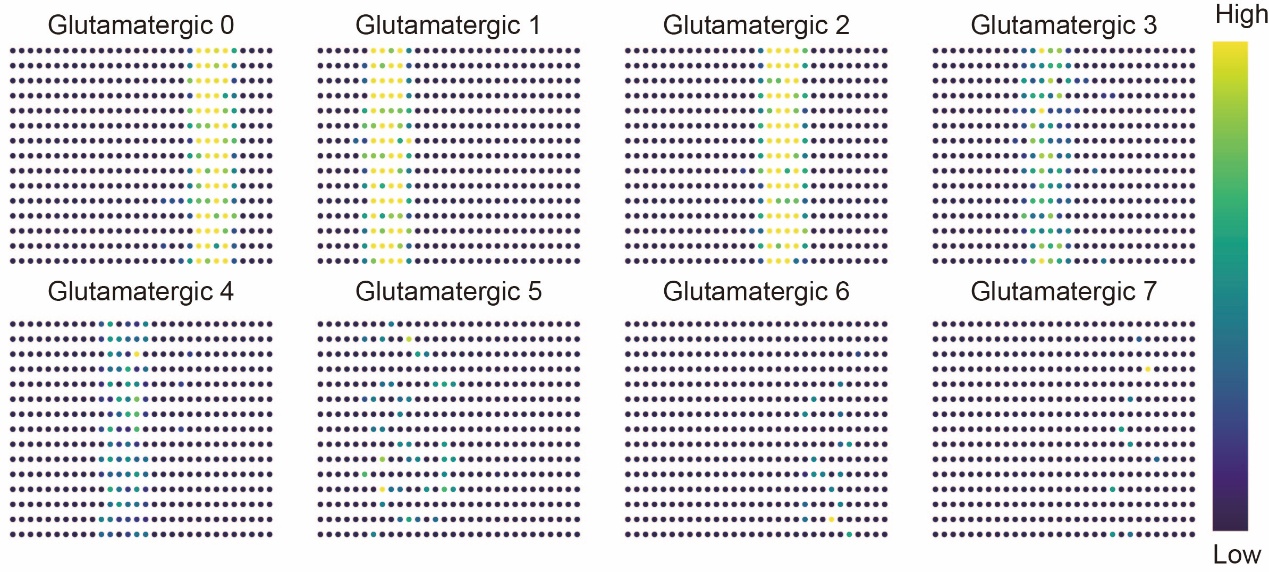


**Figure S1.** Heatmap shows the spatially specific distribution patterns of 8 glutamatergic neuronal subclusters defined by HIDF through hierarchical structure.


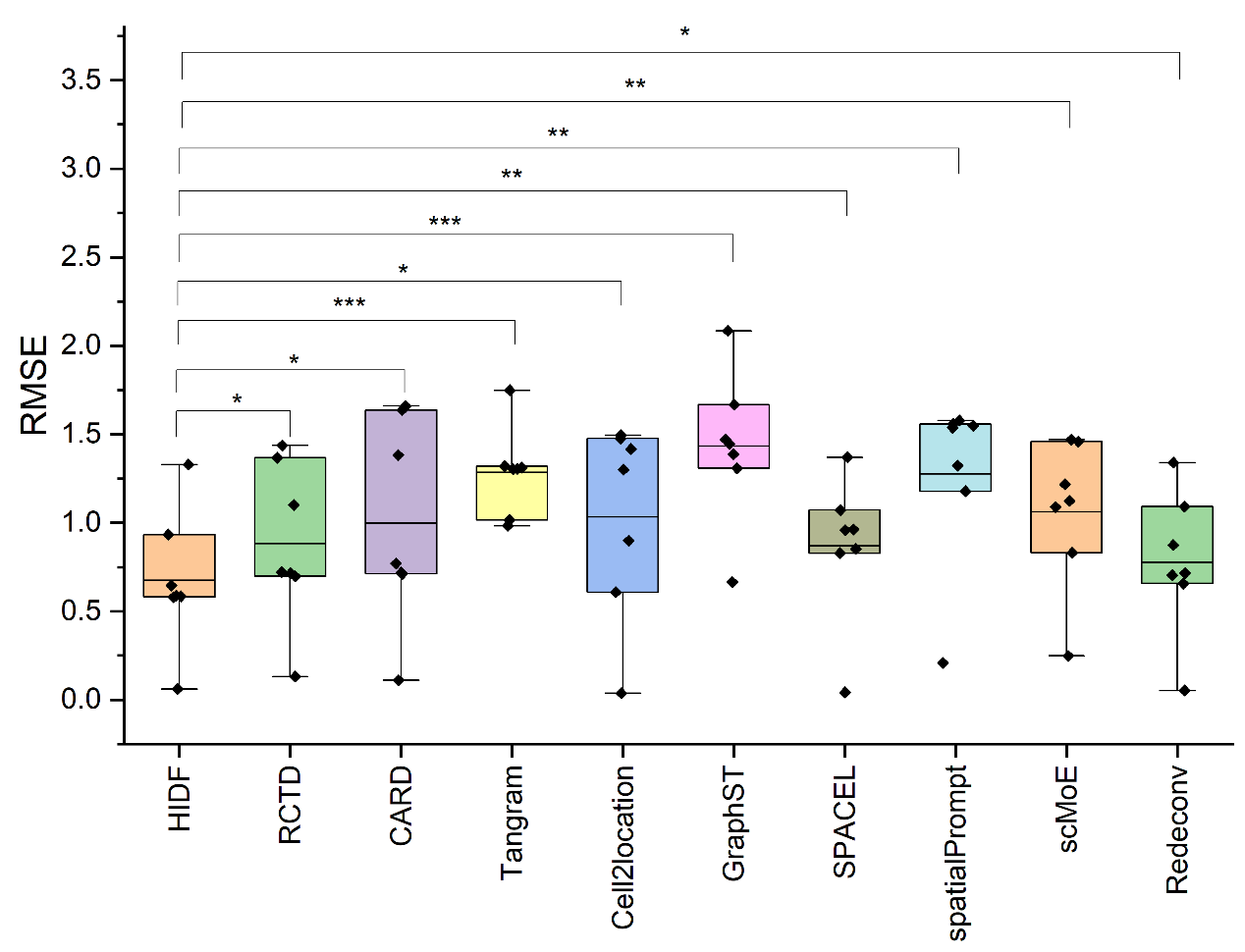


**Figure S2.** Inter-group comparisons were performed using paired one-tailed t-tests. Data are presented as box plots (box: 25th–75th percentiles; whiskers: 1.5×IQR range). Each group includes 7 results, corresponding to the RMSE (root mean square error) values of 7 independent simulated datasets (lower RMSE indicates better prediction performance). The horizontal line represents the mean value for each group (n = 7 per group). Significance is denoted as follows: *P < 0.05, **P < 0.01, ***P < 0.001.
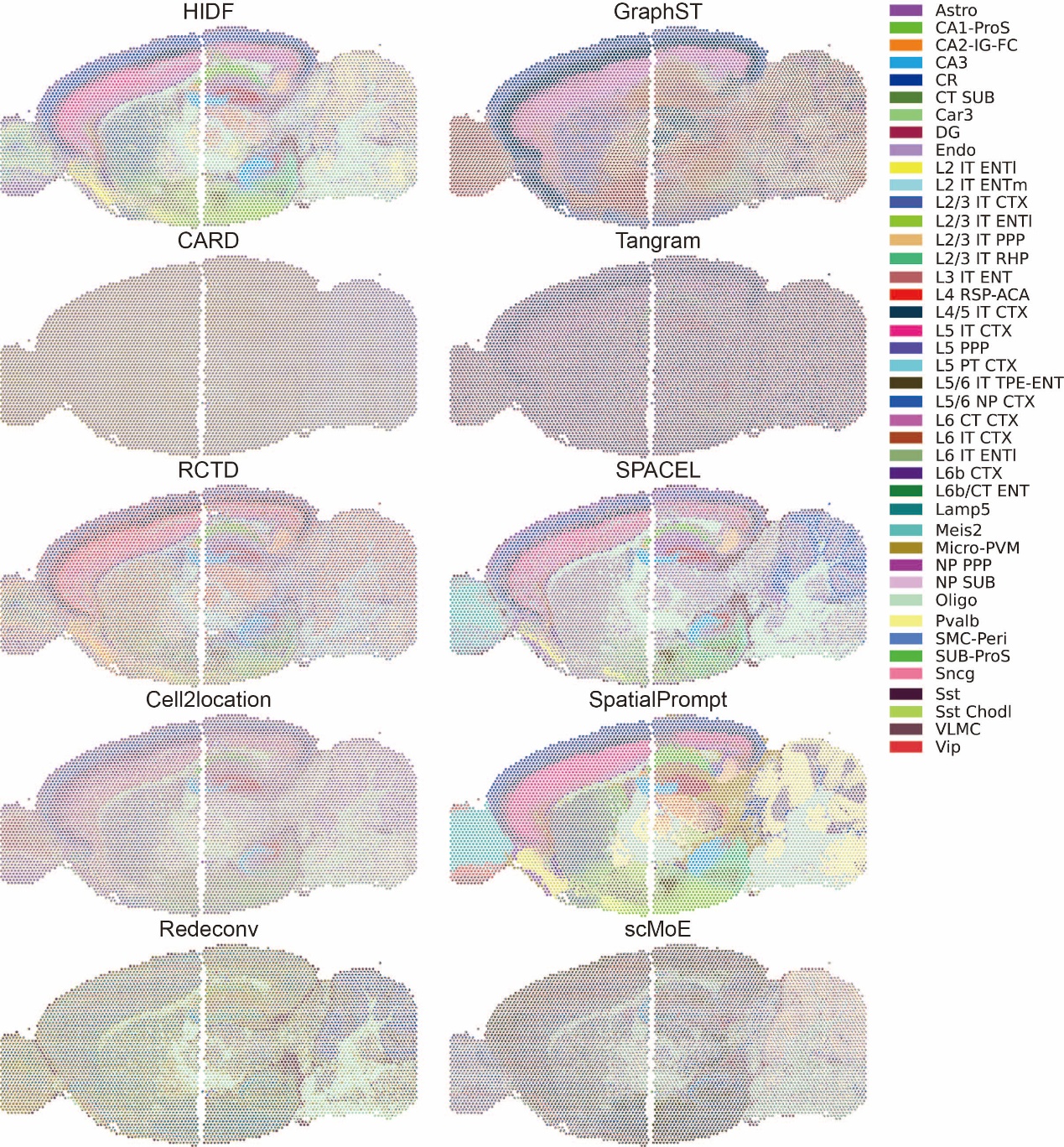


**Figure S3.** Scatter pie plot displays the spatial distribution of cell type proportions estimated by HIDF and all comparative methods.


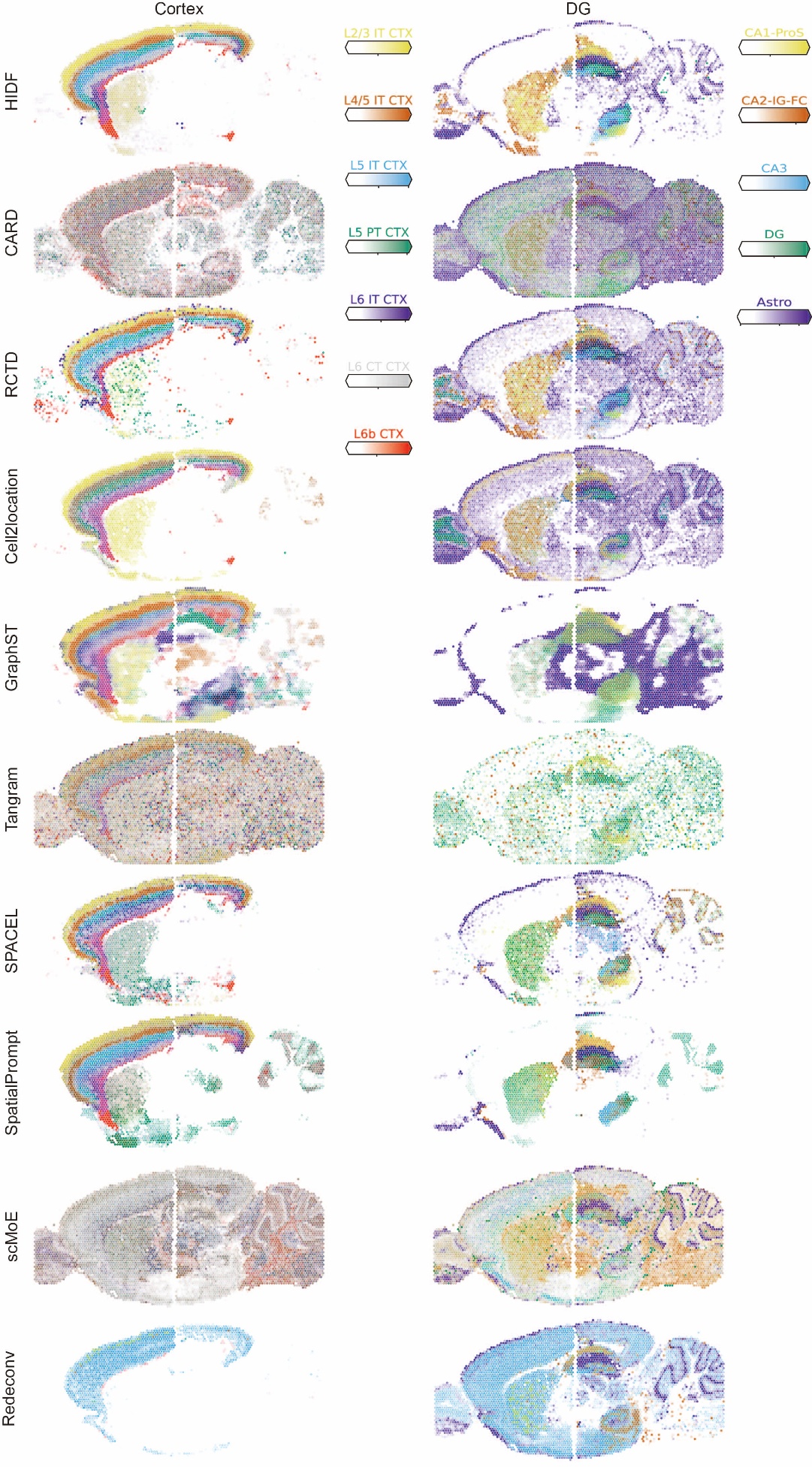


**Figure S4.** Spatial abundance distribution of cortex-related neuronal cells and dentate gyrus-related cells in the mouse brain estimated by all methods.


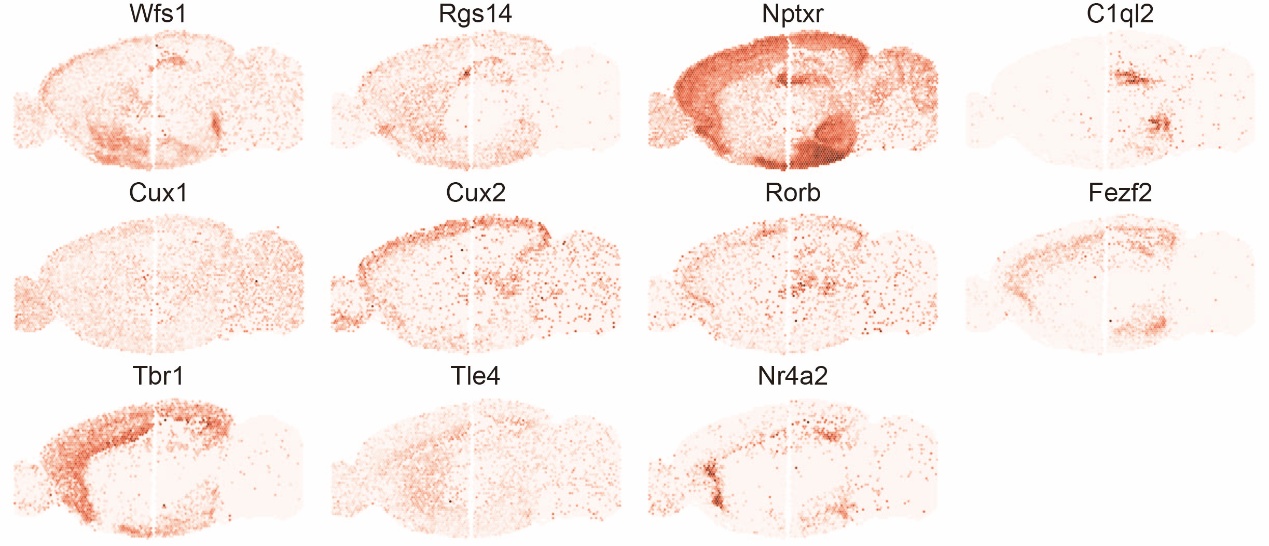


**Figure S5.** Expression heatmap of cell-type marker genes in the mouse brain dataset.


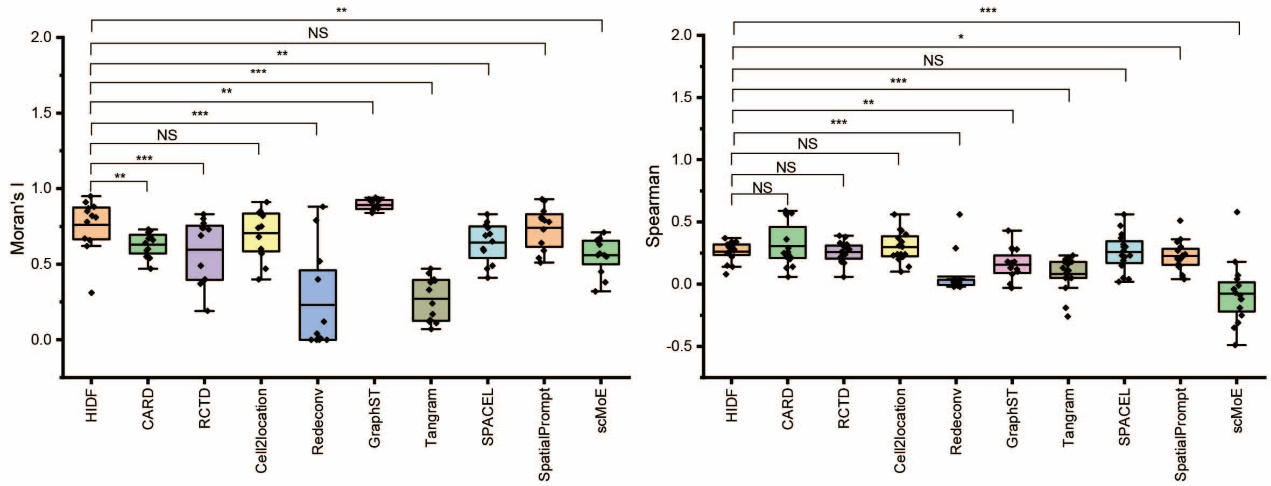


**Figure S6.** Inter-group comparisons were performed using paired one-tailed t-tests in the mouse anterior and posterior brain datasets. Data are presented as box plots (box: 25th–75th percentiles; whiskers: 1.5×IQR range). The left box plot corresponds to Moran‘s I (each group includes 12 results, n=12 per group) and the other to Spearman’s correlation coefficient (each group includes 16 results, n=16 per group); higher values of both indicators indicate better performance. The horizontal line represents the mean value for each group. Significance is denoted as follows: NS P>0.05,*P < 0.05, **P < 0.01, ***P < 0.001.
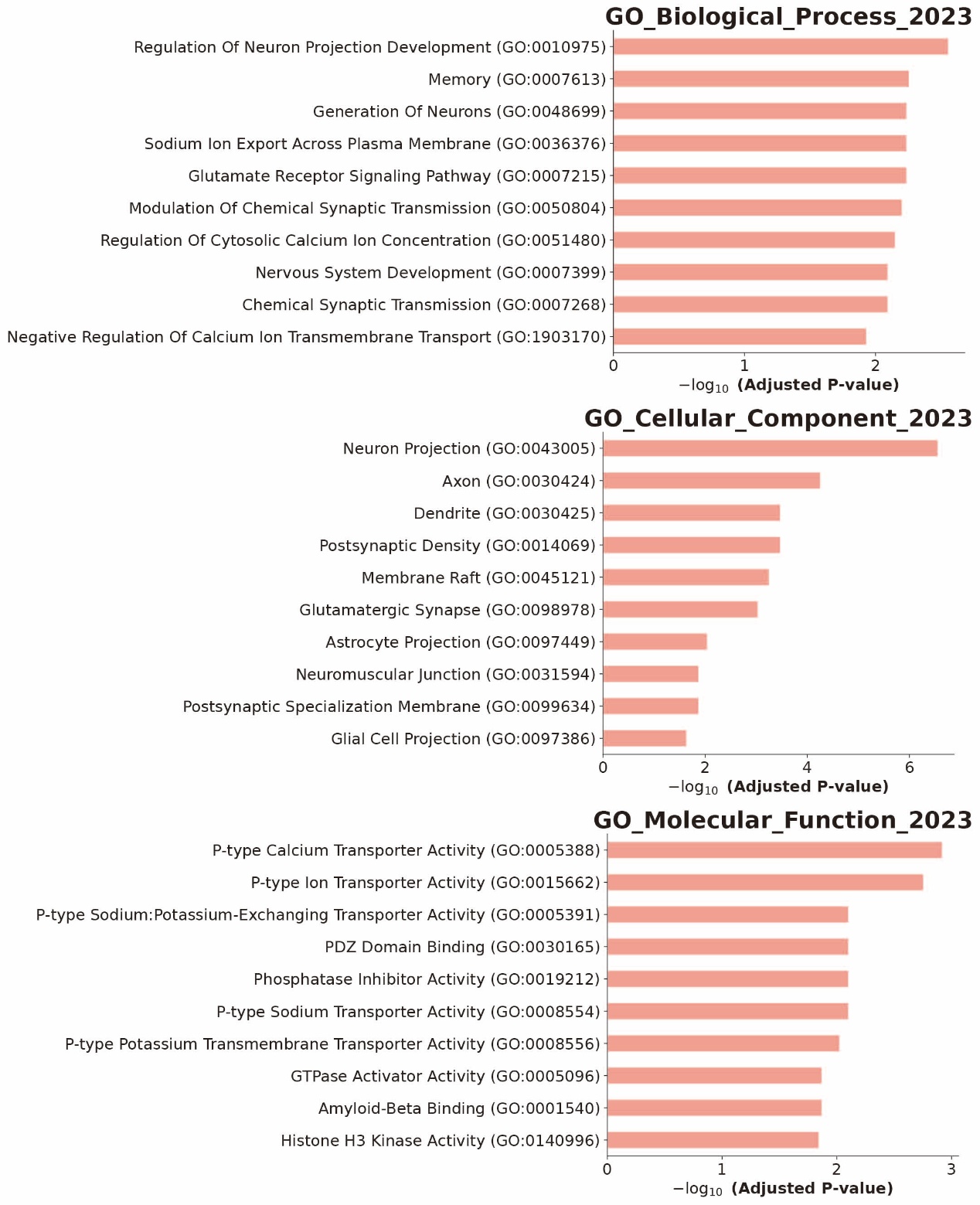


**Figure S7.** GO enrichment analysis results of astrocyte subtype 1 in the mouse pre - and post brain datasets.


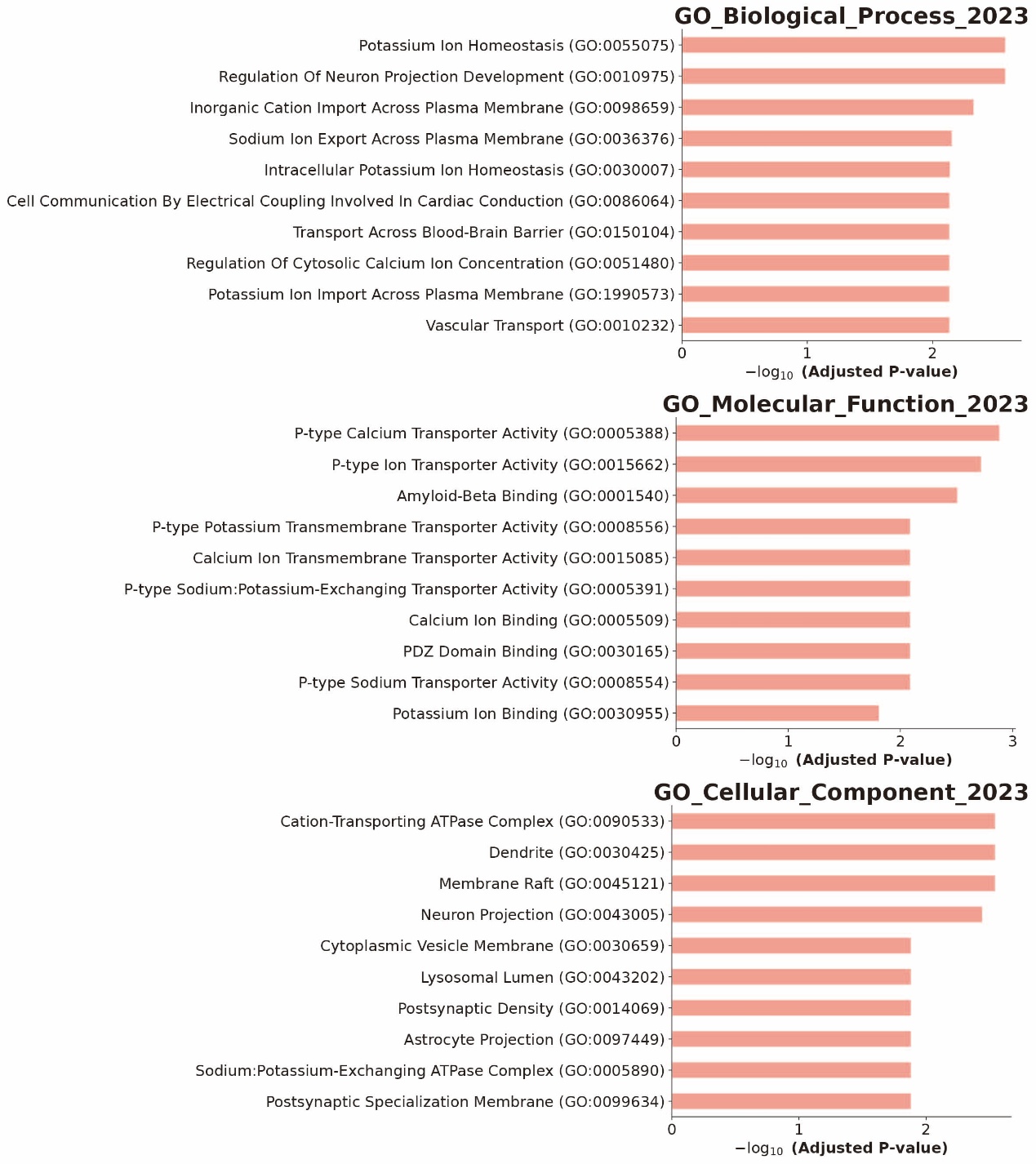


**Figure S8.** GO enrichment analysis results of astrocyte subtype 2 in the mouse anterior and posterior brain datasets.


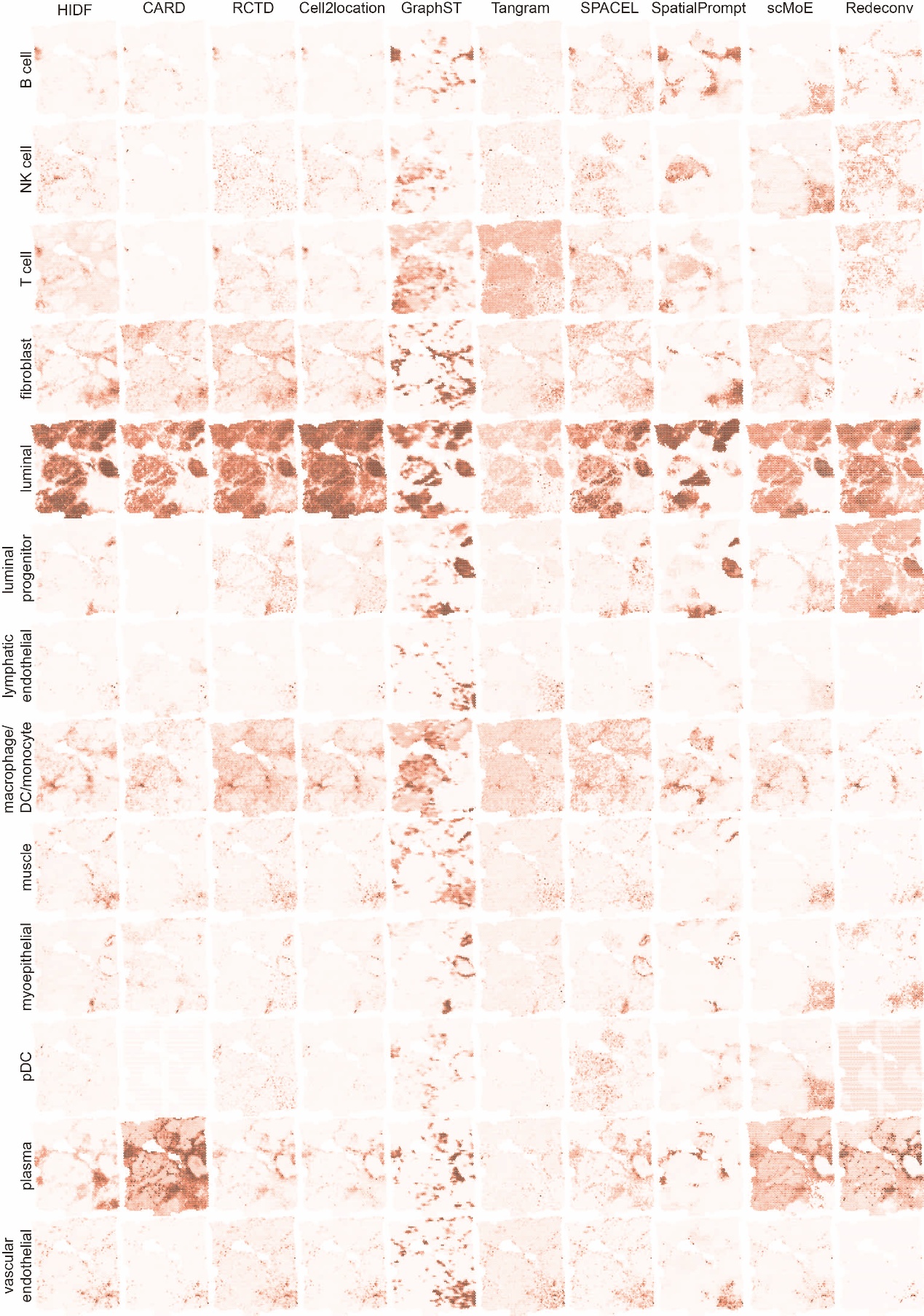


**Figure S9.** Cell type abundance heatmaps for all comparative methods in the human breast cancer dataset.


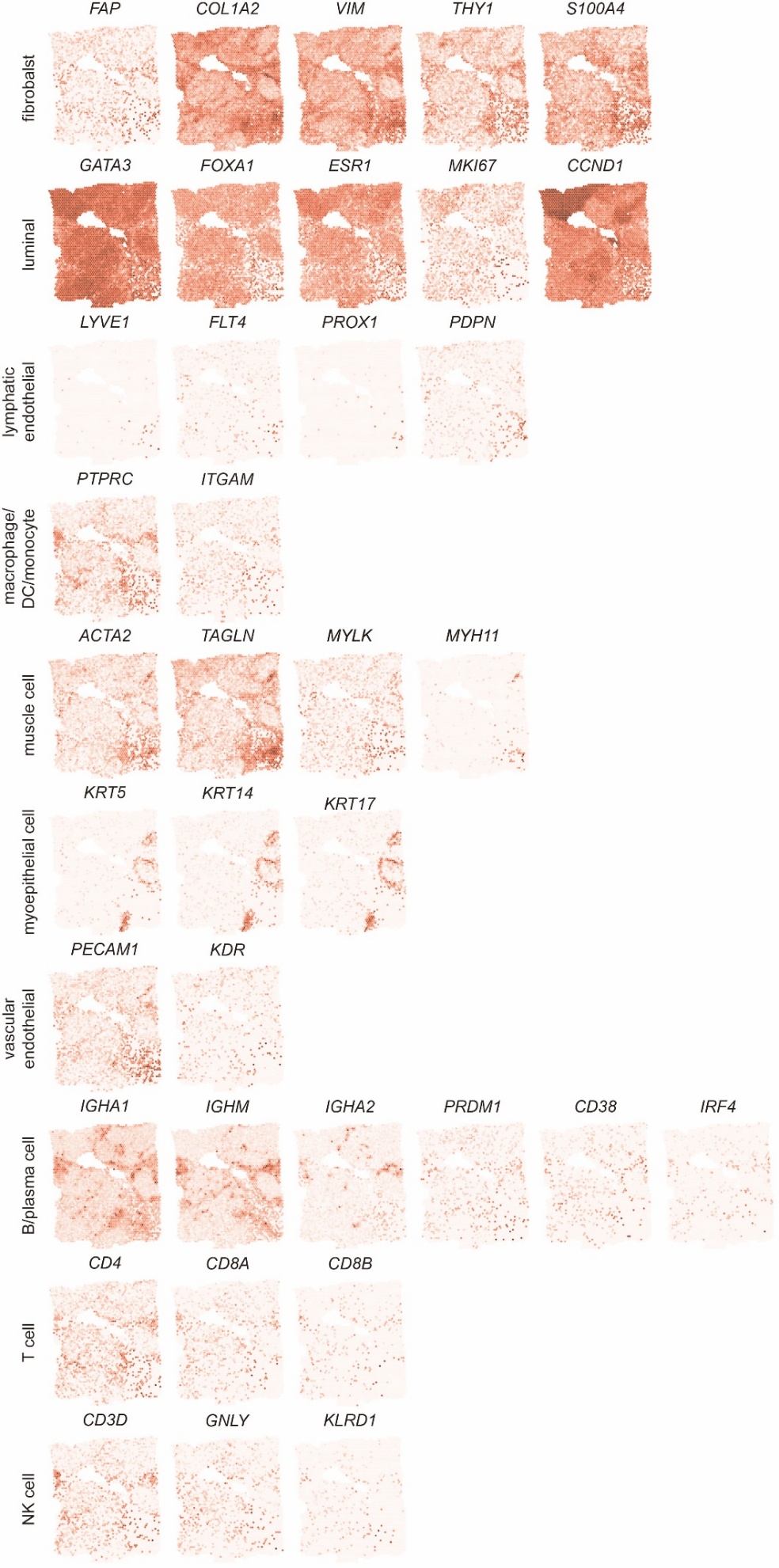


**Figure S10.** Expression heatmap of cell-type marker genes in the human breast cancer dataset.


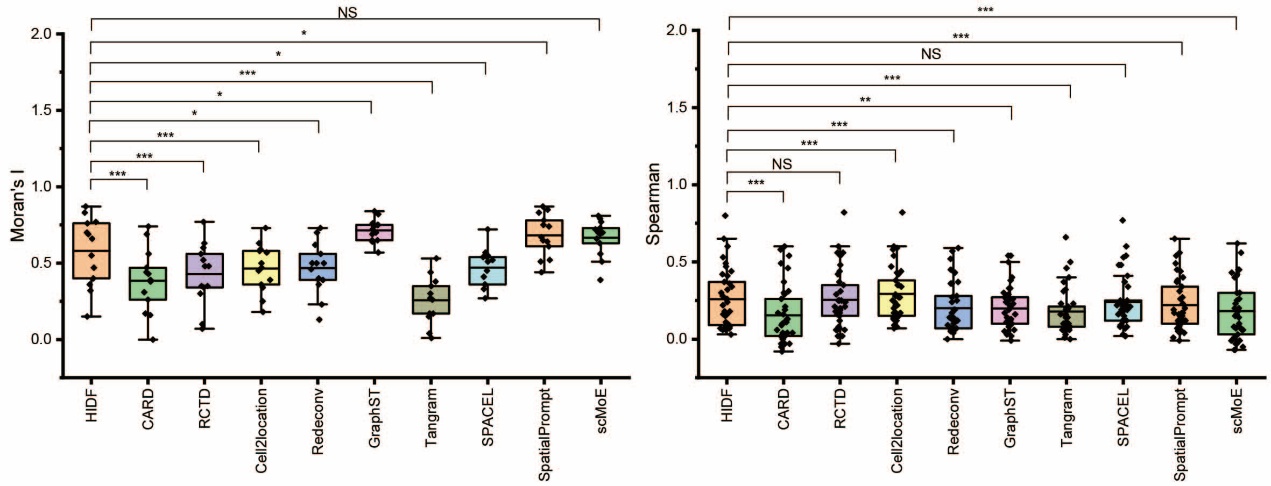


**Figure S11.** Inter-group comparisons were performed using paired one-tailed t-tests in the human breast cancer dataset. Data are presented as box plots (box: 25th–75th percentiles; whiskers: 1.5×IQR range). The left box plot corresponds to Moran‘s I (each group includes 13 results, n=13 per group) and the other to Spearman’s correlation coefficient (each group includes 37 results, n=37 per group); higher values of both indicators indicate better performance. The horizontal line represents the mean value for each group. Significance is denoted as follows: NS P>0.05,*P < 0.05, **P < 0.01, ***P < 0.001.
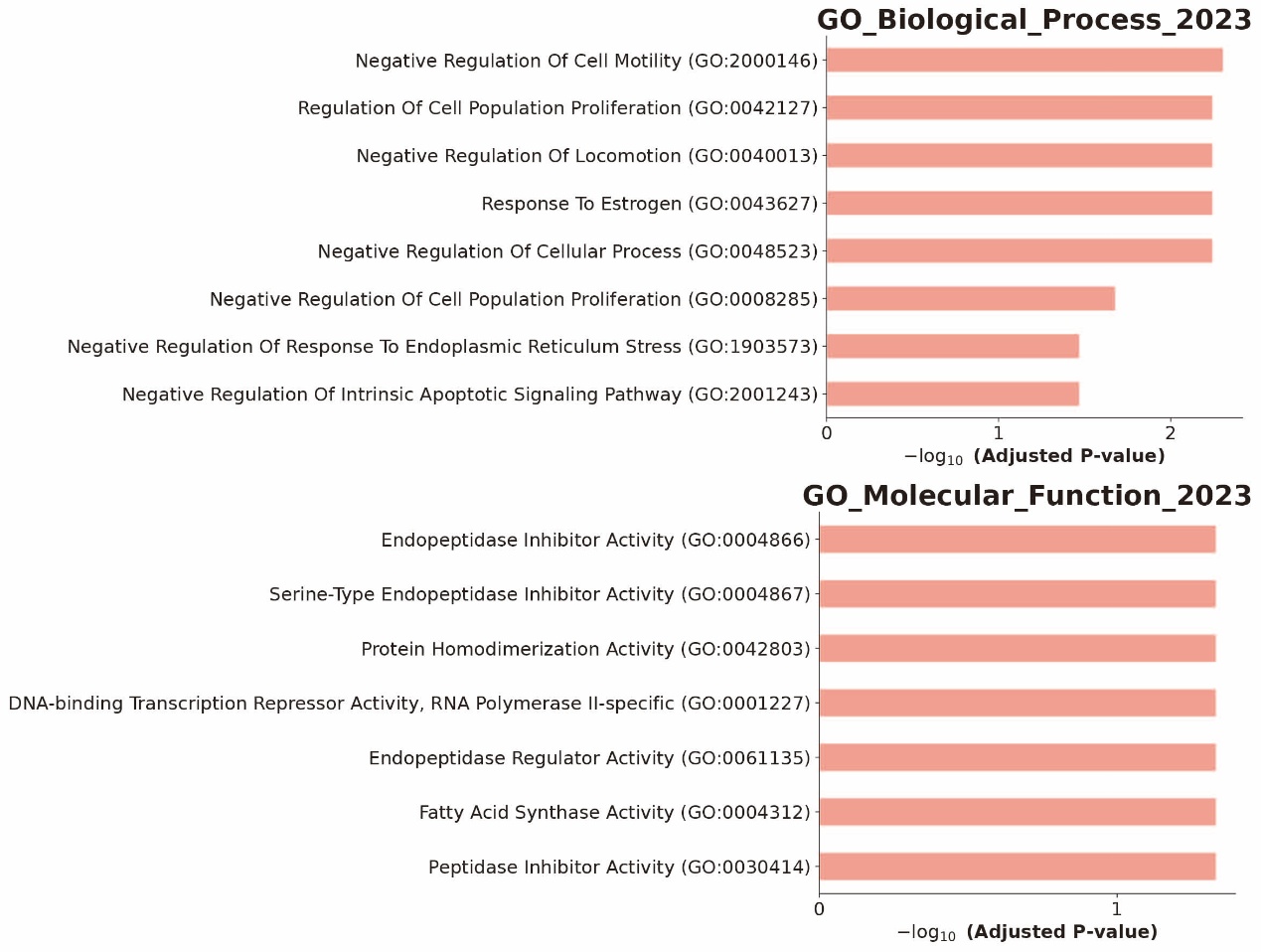


**Figure S12.** GO enrichment analysis results of luminal A subtype cells in human breast cancer dataset.


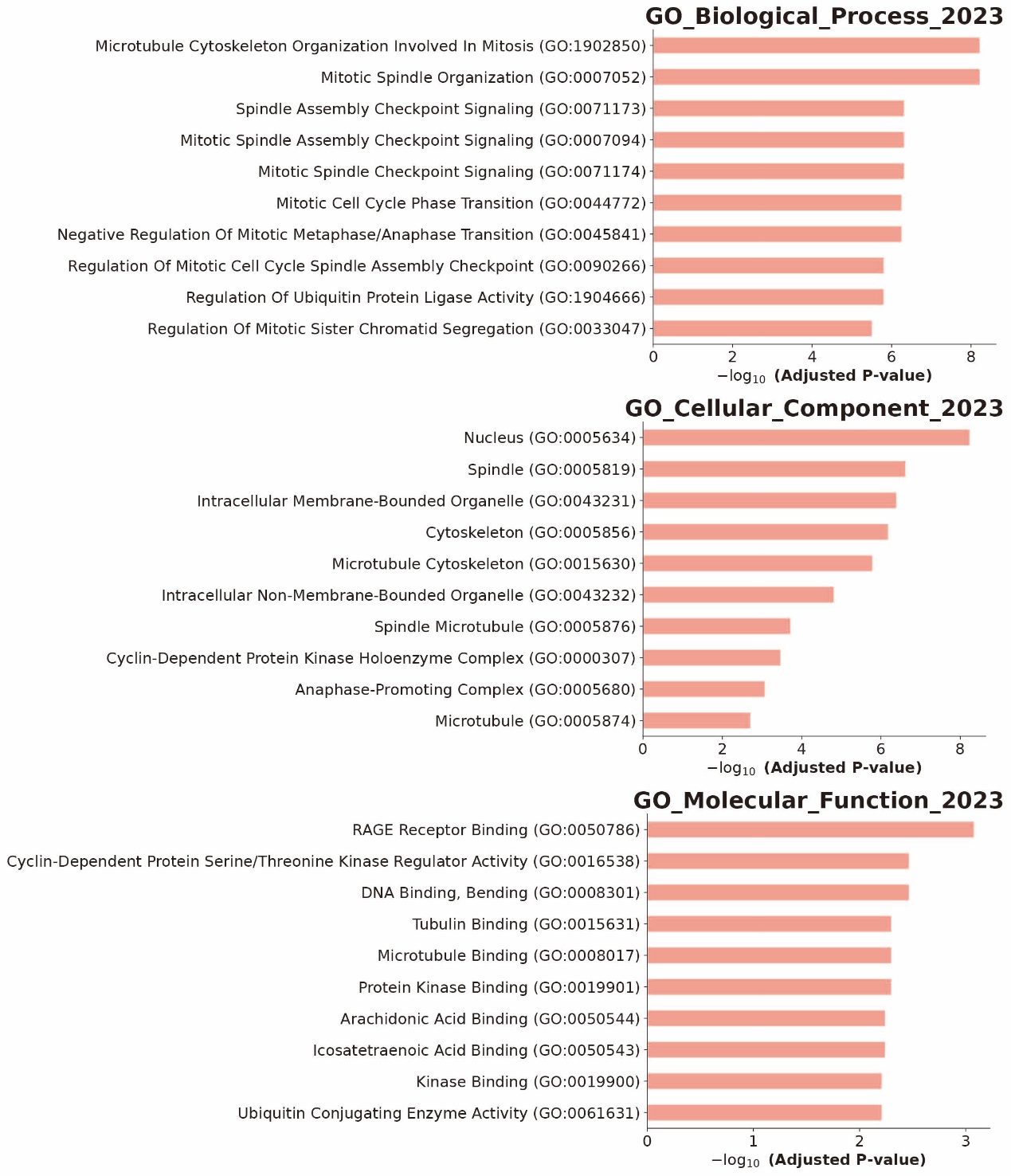


**Figure S13.** GO enrichment analysis results of luminal B subtype cells in human breast cancer dataset.


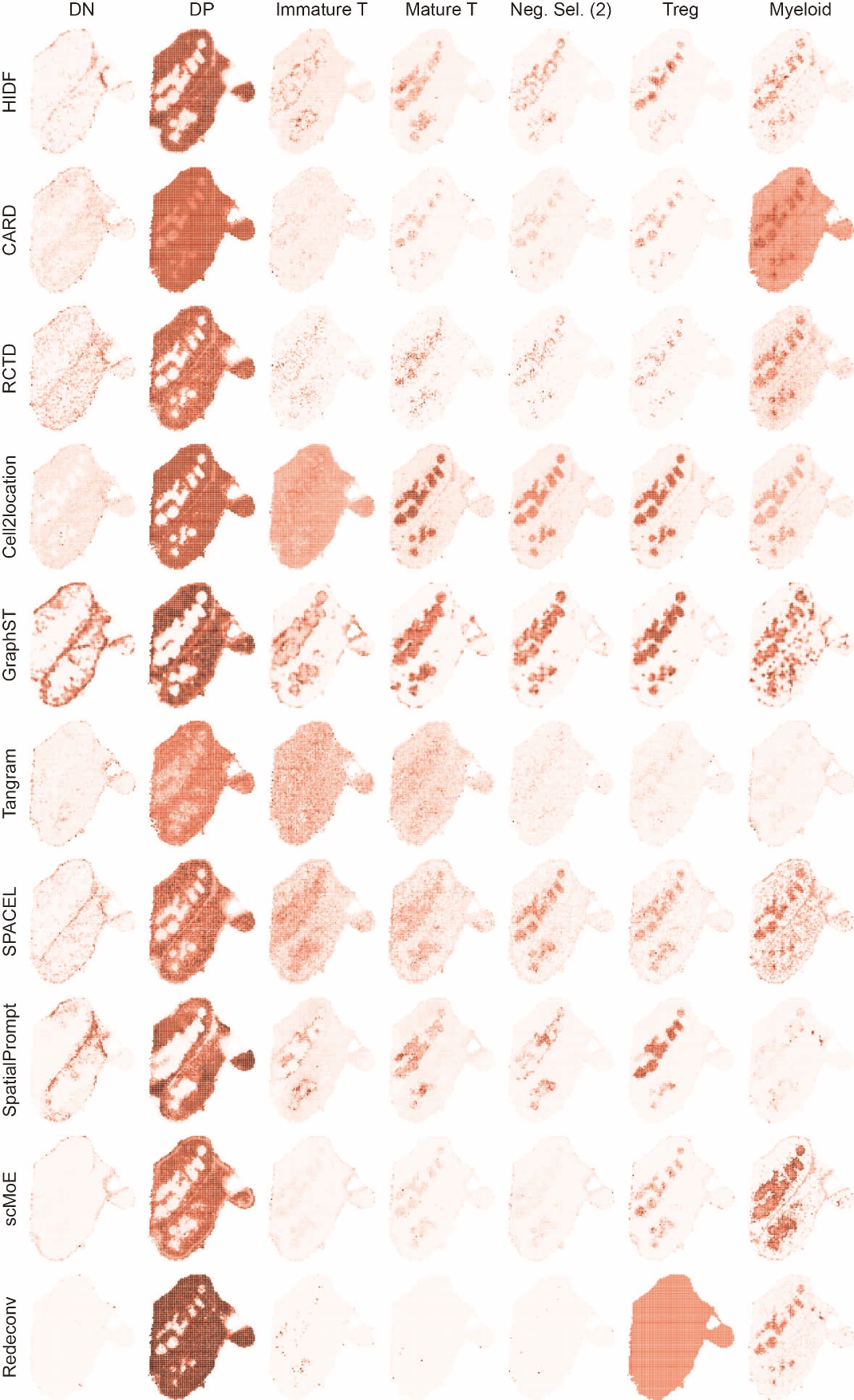


**Figure S14.** Cell type abundance heatmaps of T cells at distinct developmental stages and myeloid cells are estimated by all comparative methods.


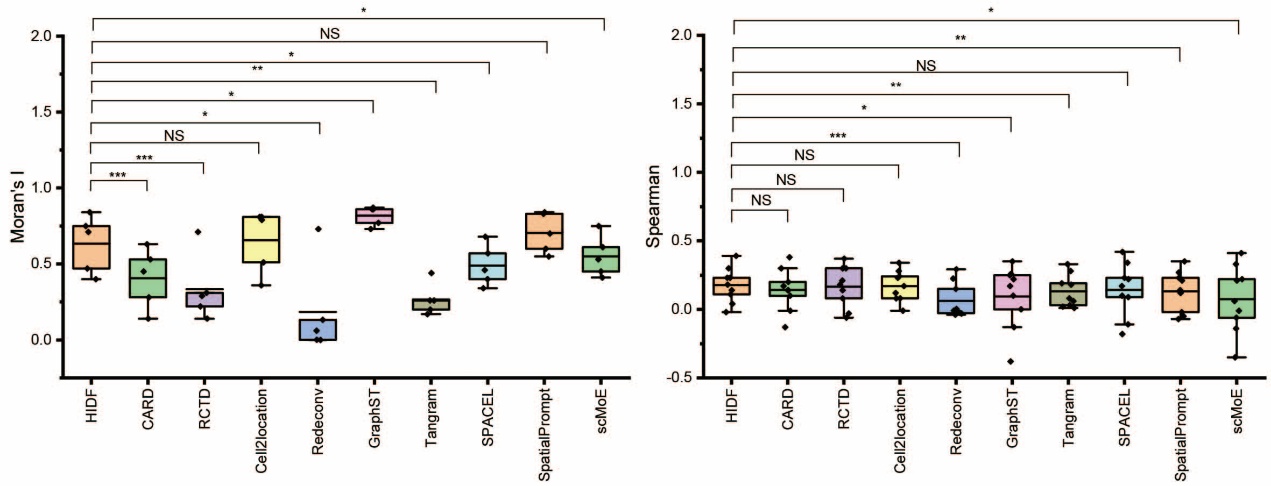


**Figure S15.** Inter-group comparisons were performed using paired one-tailed t-tests in the mouse thymus dataset. Data are presented as box plots (box: 25th–75th percentiles; whiskers: 1.5×IQR range). The left box plot corresponds to Moran‘s I (each group includes 5 results, n=5 per group) and the other to Spearman’s correlation coefficient (each group includes 9 results, n=9 per group); higher values of both indicators indicate better performance. The horizontal line represents the mean value for each group. Significance is denoted as follows: NS P>0.05, *P < 0.05, **P < 0.01, ***P < 0.001.


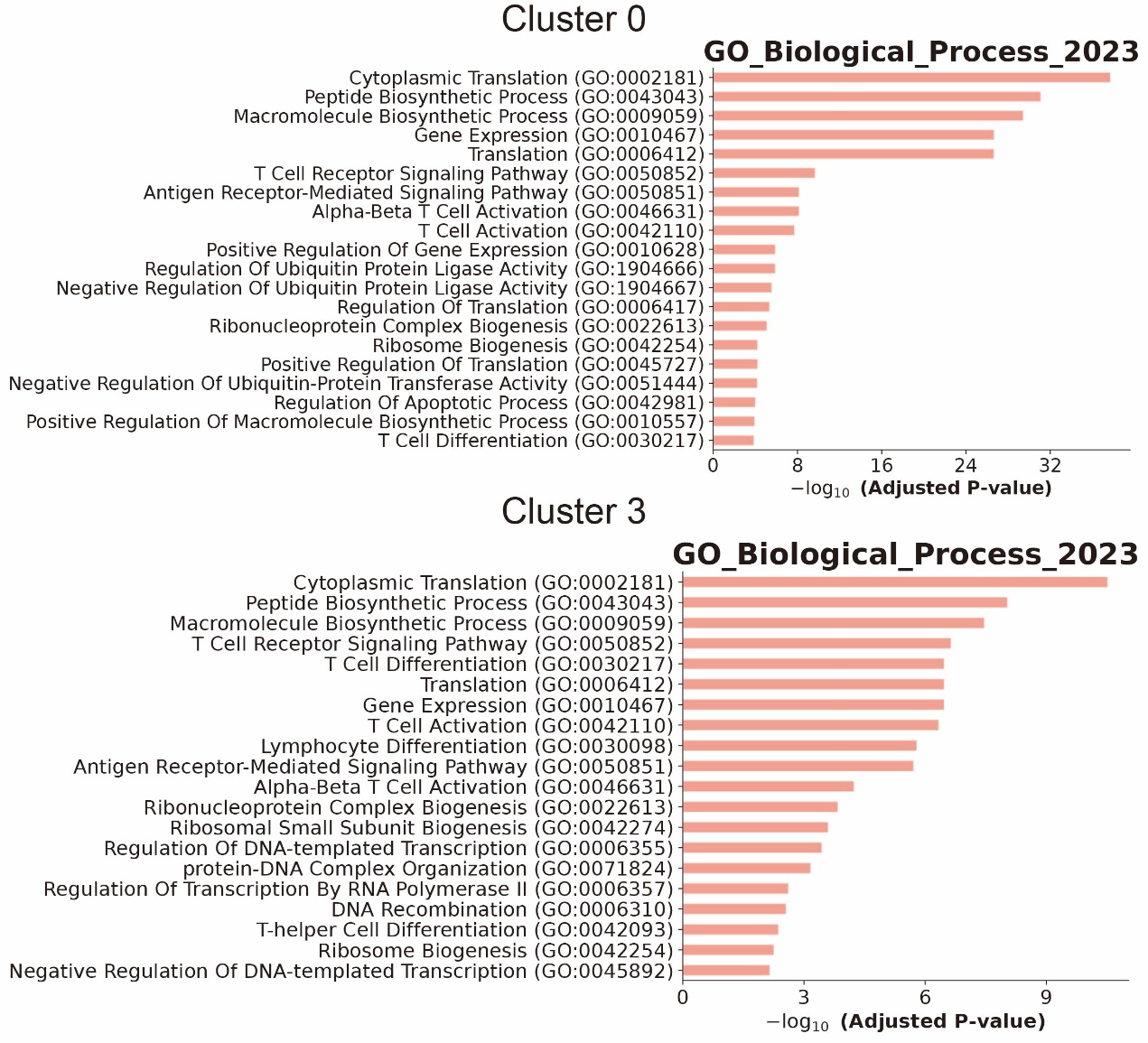


**Figure S16.** GO Biological Process enrichment results of myeloid sub cluster 0 and 3.


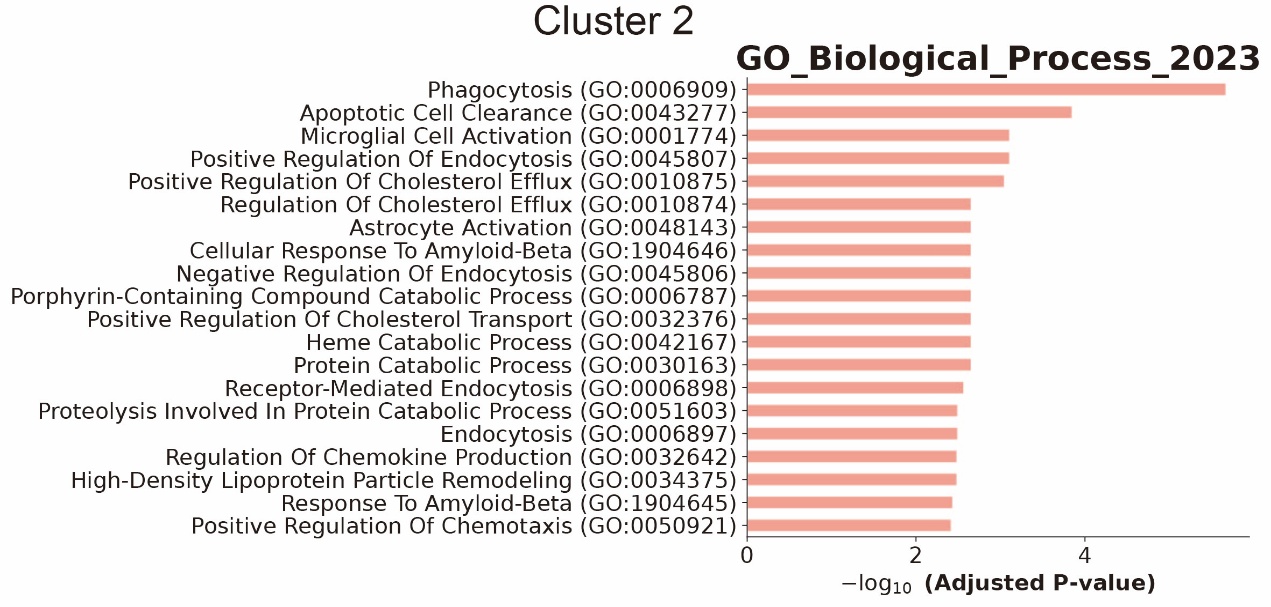


**Figure S17.** GO Biological Process enrichment results of myeloid sub cluster 2.


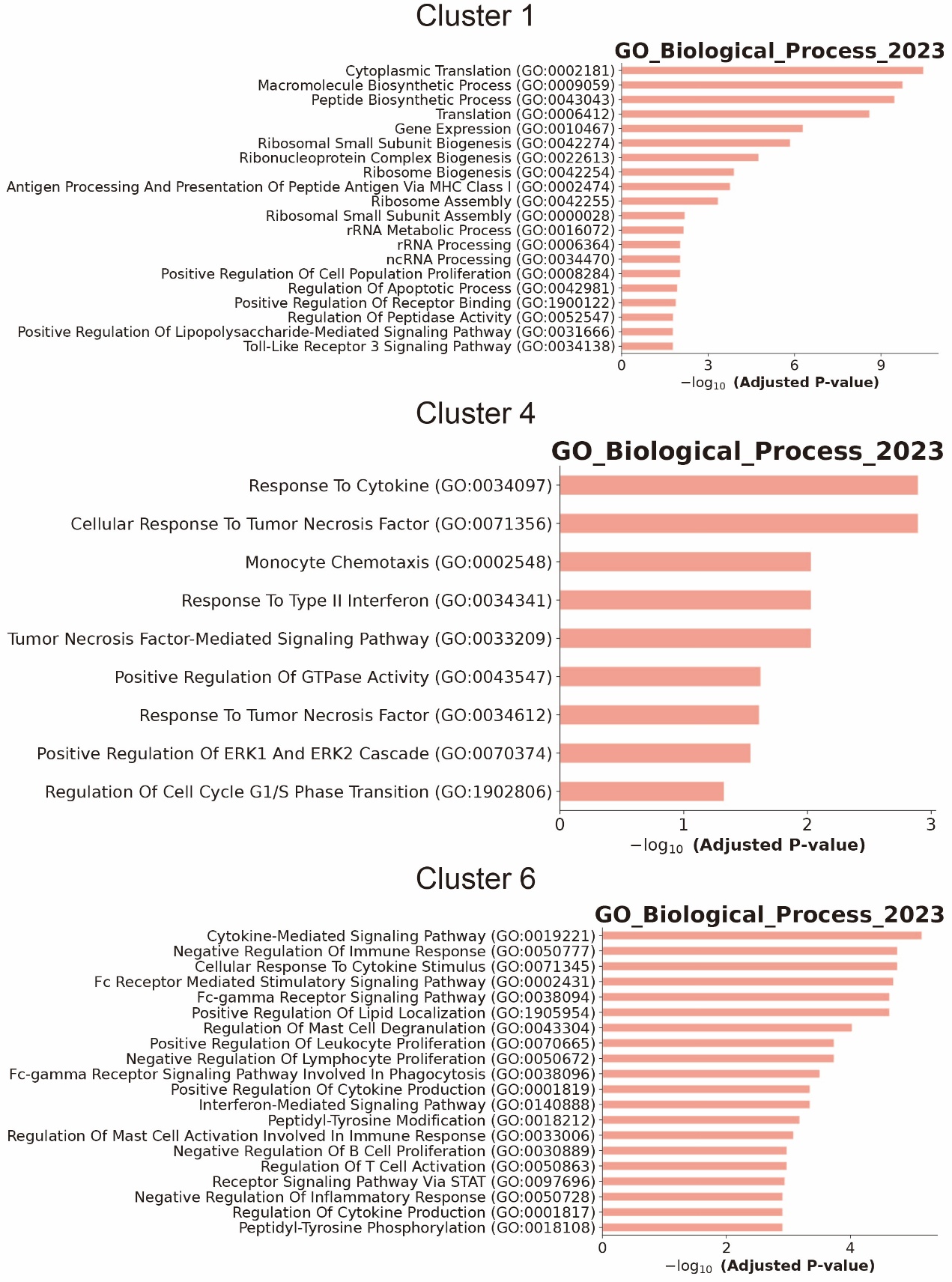


**Figure S18.** GO Biological Process enrichment results of myeloid sub cluster 1, 4 and 6.


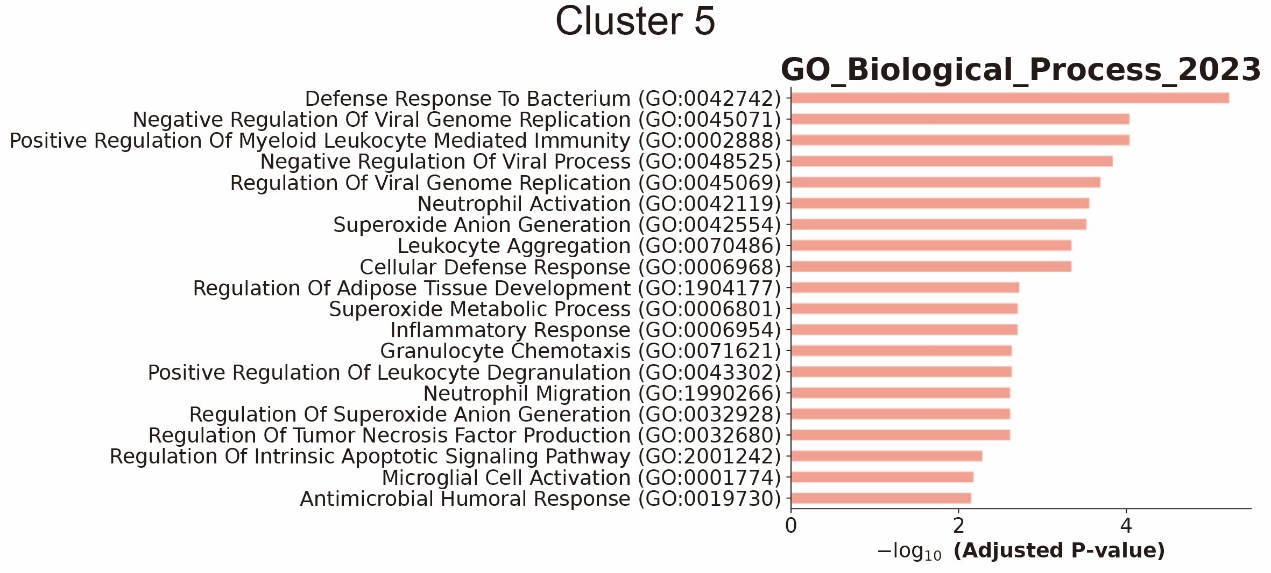


**Figure S19.** GO Biological Process enrichment results of Myeloid sub types.


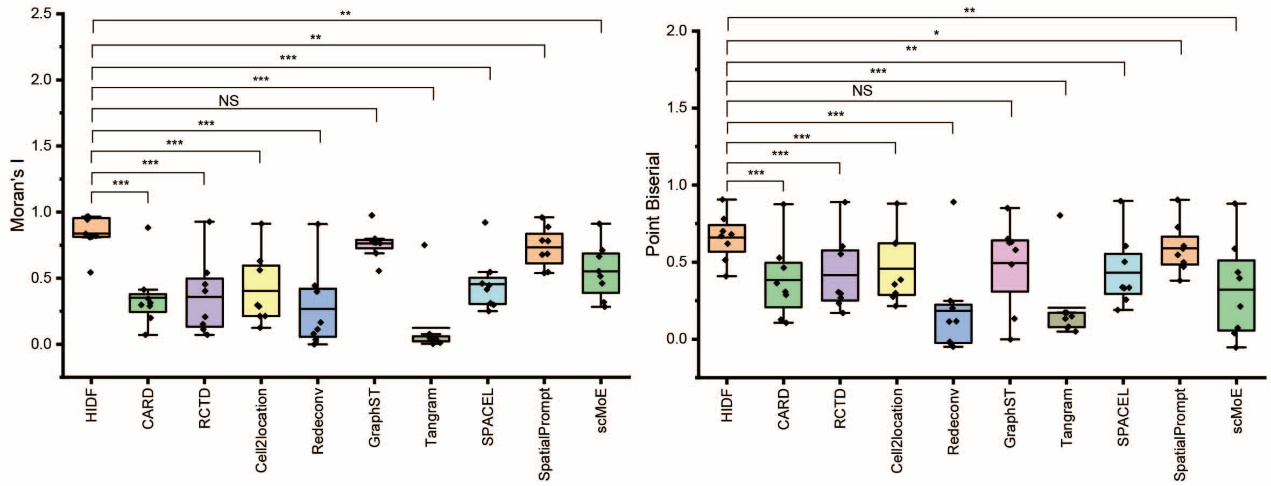


**Figure S20.** Inter-group comparisons were performed using paired one-tailed t-tests in the human dlpfc dataset. Data are presented as box plots (box: 25th–75th percentiles; whiskers: 1.5×IQR range). The left box plot corresponds to Moran‘s I (each group includes 8 results, n=8 per group) and the other to Point Biserial (each group includes 8 results, n=8 per group); higher values of both indicators indicate better performance. The horizontal line represents the mean value for each group. Significance is denoted as follows: NS P>0.05,*P < 0.05, **P < 0.01, ***P < 0.001.


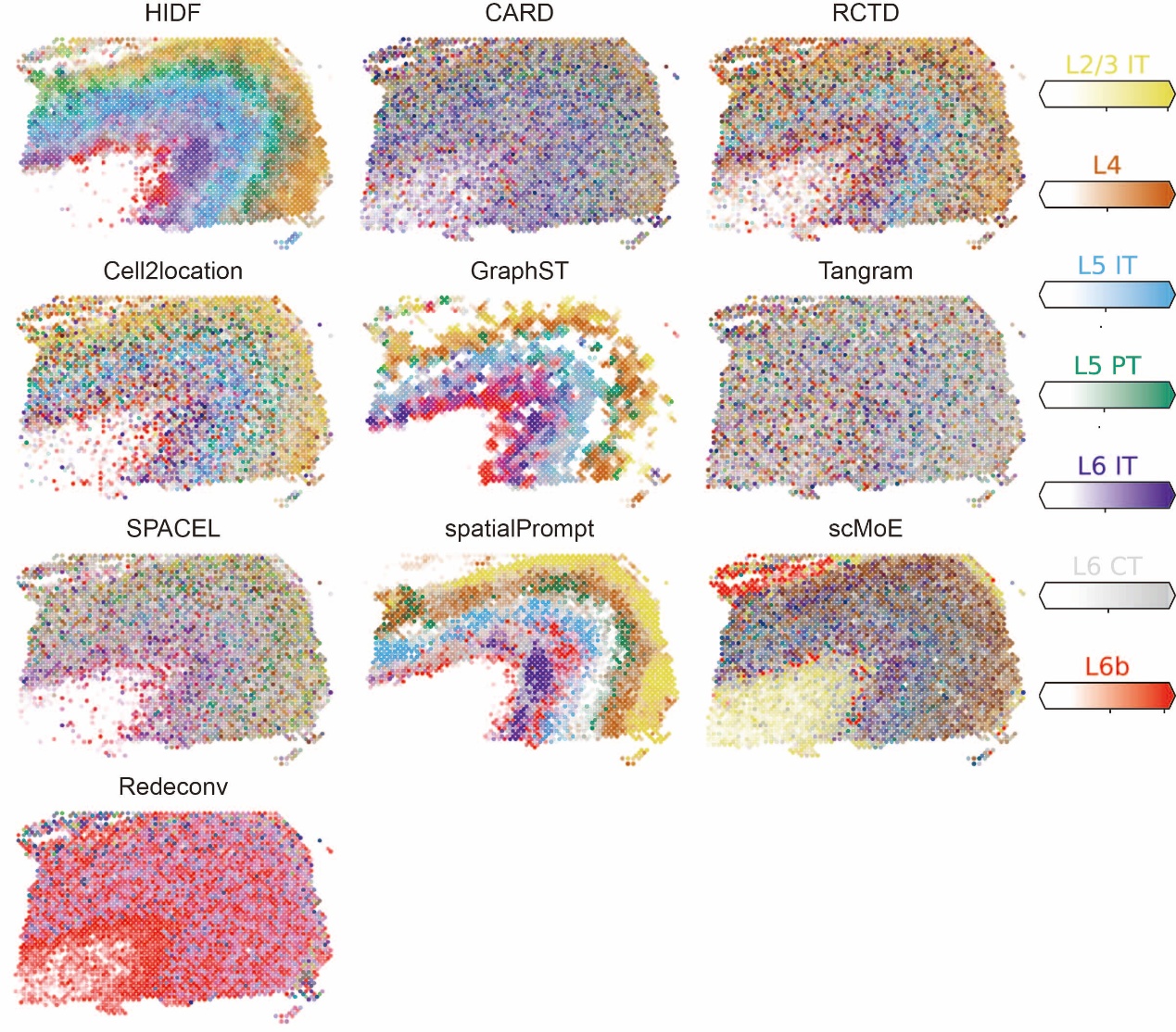


**Figure S21.** Heatmap of neuronal subtype distribution estimated by all methods in the human DLPFC dataset.


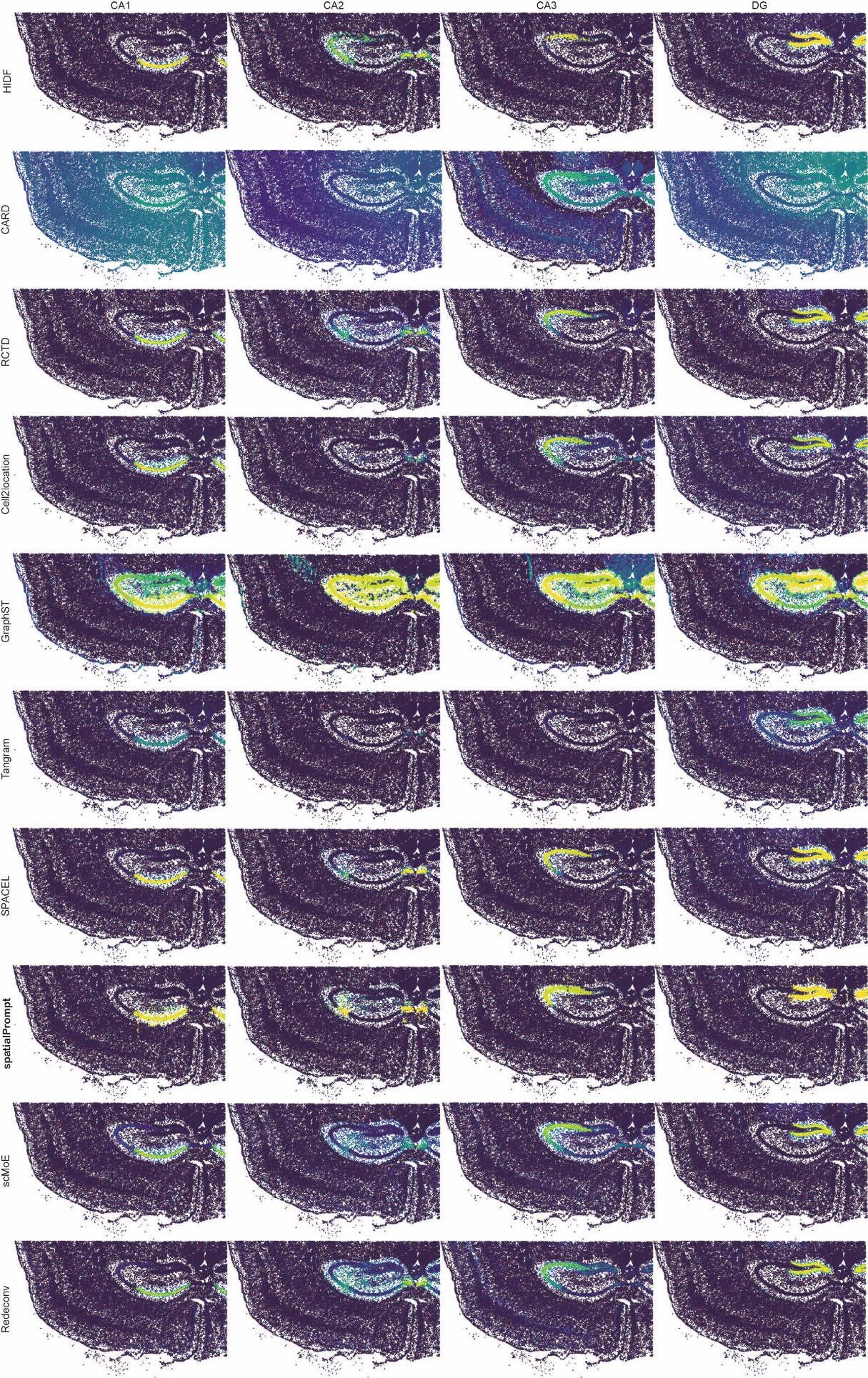


**Figure S22.** Heatmaps of DG, CA1, CA2, and CA3 cell types in the deconvolution results of all methods in the mouse brain Xenium dataset.


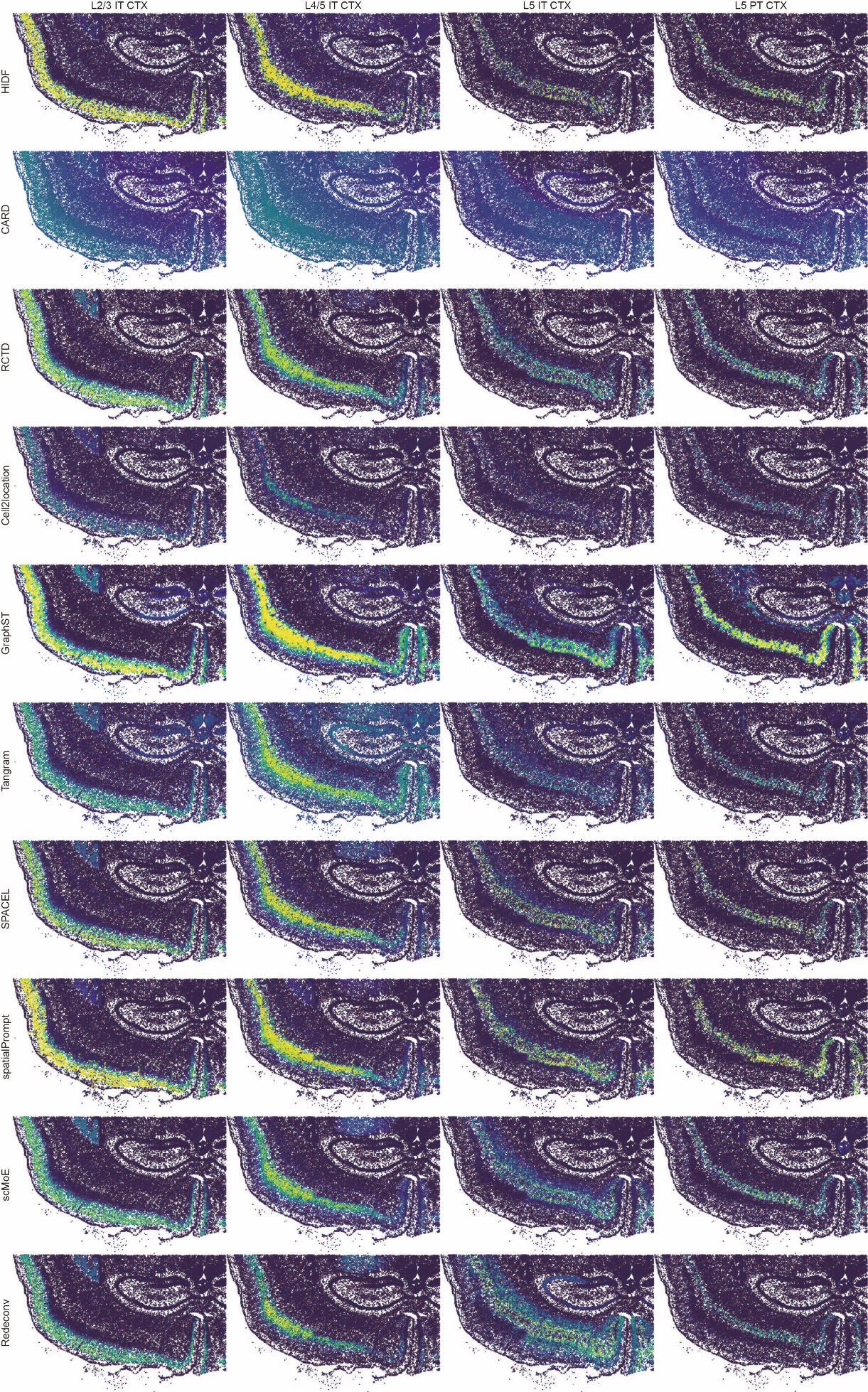


**Figure S23.** Heatmaps of L2/3 IT, L4/5 IT, L5 IT, and L5 PT cell types in the deconvolution results of all methods in the mouse brain Xenium dataset.


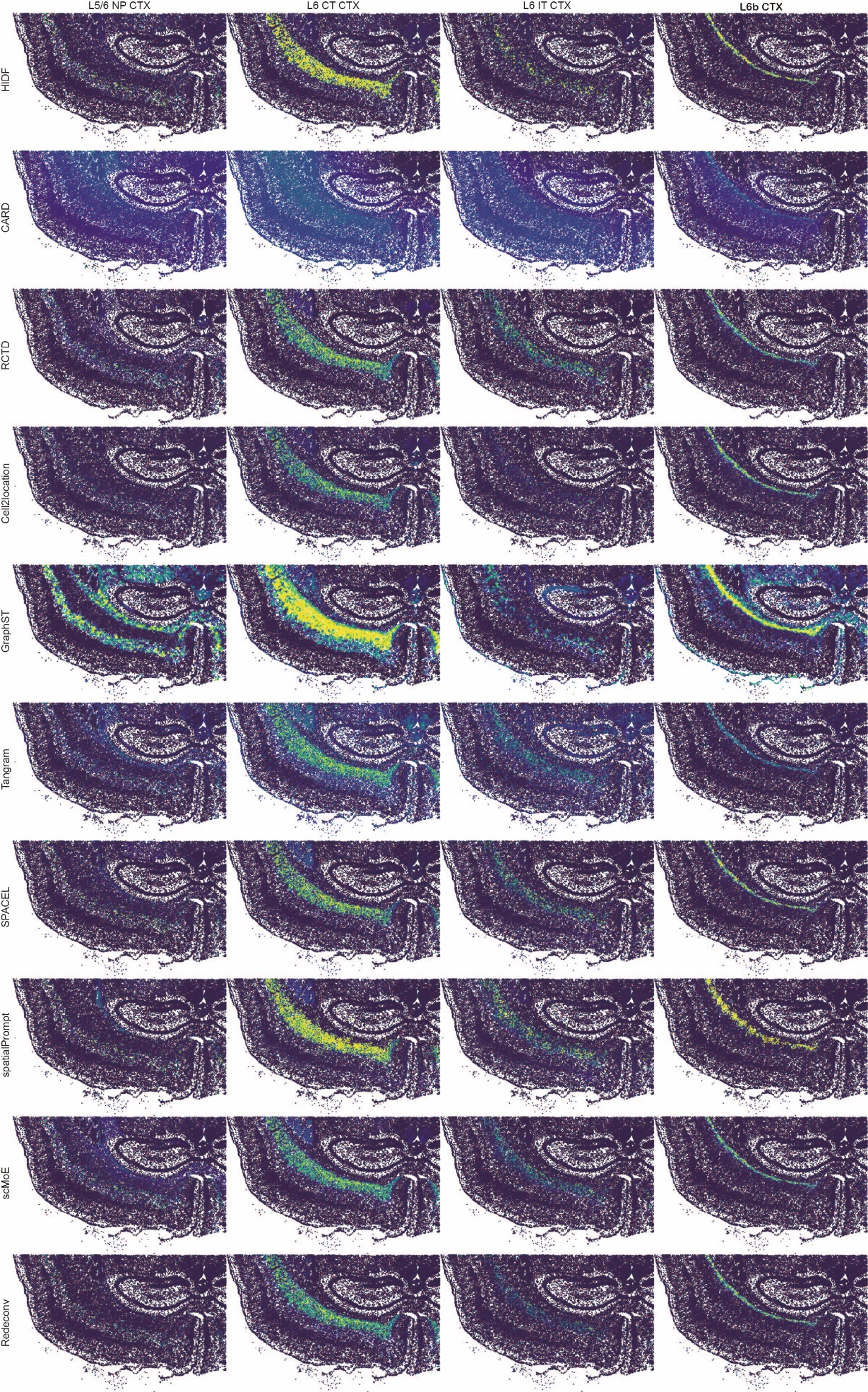


**Figure S24.** Heatmaps of L5/6 NP, L6 CT, L6 IT, and L6b cell types in the deconvolution results of all methods in the mouse brain Xenium dataset.


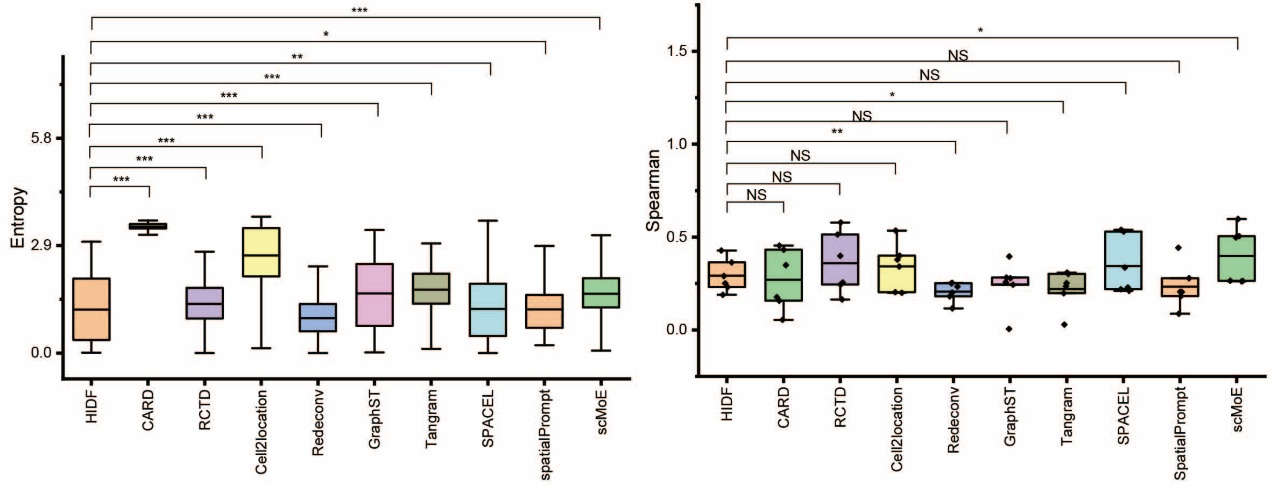


**Figure S25.** Inter-group comparisons were performed using paired one-tailed t-tests in the mouse brain Xenium dataset. Data are presented as box plots (box: 25th–75th percentiles; whiskers: 1.5×IQR range). The left box plot corresponds to Entropy (each group includes 36532 results, n=36532 per group) and the other to Spearman’s correlation coefficient (each group includes 6 results, n=6 per group); higher values of both indicators indicate better performance. The horizontal line represents the mean value for each group. Significance is denoted as follows: NS P>0.05, *P < 0.05, **P < 0.01, ***P < 0.001.


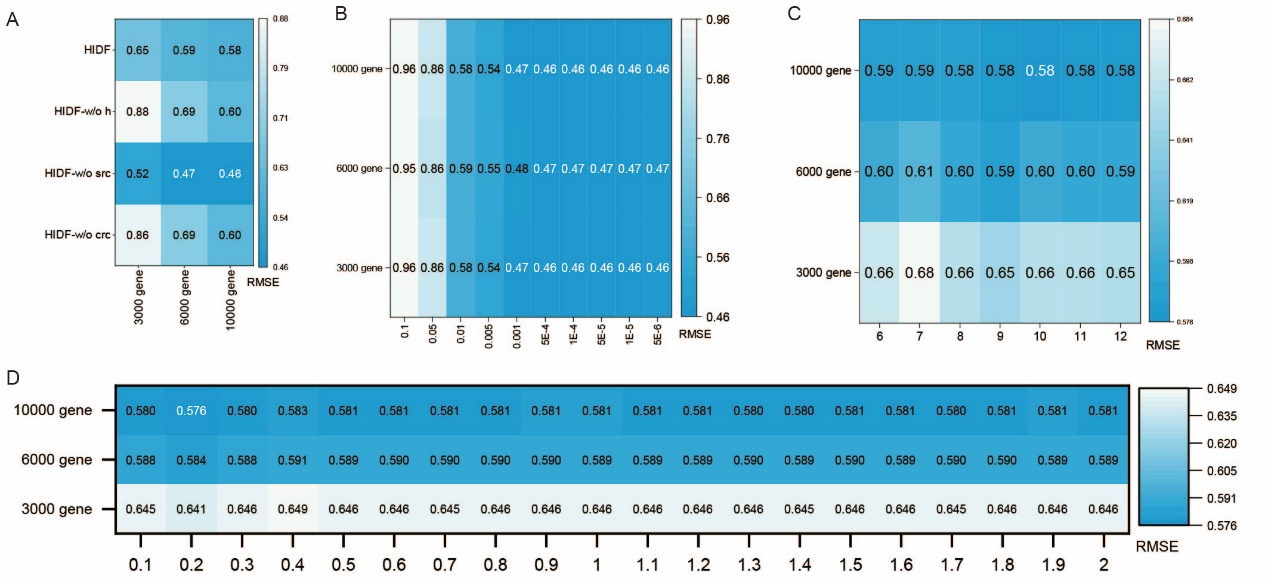


**Figure S26.** Results of ablation experiments and parameter analysis on seqFISH dataset. (A) Ablation analysis of different modules. (B) Effect of the spatial regularization parameter λ on model performance. (C) Effect of the neighbor count k on model performance. (D) Effect of the Leiden resolution parameter on model performance.

**Table S1.** Root mean square error (RMSE) between deconvolution results and ground truth cell type distributions in the seqFISH3000 dataset, including RMSE per cell type and the summed RMSE across all cell types. Values are rounded to two decimal places.

|  | HIDF | RCTD | CARD | Cell2location | Redeconv | Tangram | GraphST | SPACEL | SpatialPrompt | scMoE |
| --- | --- | --- | --- | --- | --- | --- | --- | --- | --- | --- |
| iNeuron | 0.13 | 0.12 | 0.11 | 0.14 | 0.11 | 0.14 | 0.15 | 0.14 | 0.27 | 0.21 |
| eNeuron | 0.16 | 0.22 | 0.25 | 0.54 | 0.21 | 0.48 | 0.52 | 0.28 | 0.5 | 0.48 |
| Olig | 0.09 | 0.08 | 0.09 | 0.11 | 0.08 | 0.36 | 0.42 | 0.11 | 0.27 | 0.21 |
| microglia | 0.06 | 0.09 | 0.08 | 0.38 | 0.07 | 0.06 | 0.07 | 0.05 | 0.09 | 0.24 |
| endo.mural | 0.11 | 0.09 | 0.11 | 0.13 | 0.11 | 0.14 | 0.16 | 0.1 | 0.28 | 0.19 |
| astrocytes | 0.1 | 0.11 | 0.12 | 0.12 | 0.13 | 0.12 | 0.15 | 0.17 | 0.18 | 0.15 |
| Total | 0.65 | 0.72 | 0.77 | 1.42 | 0.7 | 1.3 | 1.47 | 0.85 | 1.58 | 1.47 |

**Table S2.** Root mean square error (RMSE) between deconvolution results and ground truth cell type distributions in the seqFISH6000 dataset, including RMSE per cell type and the summed RMSE across all cell types. Values are rounded to two decimal places.

|  | HIDF | RCTD | CARD | Cell2location | Redeconv | Tangram | GraphST | SPACEL | SpatialPrompt | scMoE |
| --- | --- | --- | --- | --- | --- | --- | --- | --- | --- | --- |
| iNeuron | 0.12 | 0.1 | 0.13 | 0.14 | 0.13 | 0.14 | 0.16 | 0.12 | 0.27 | 0.17 |
| eNeuron | 0.15 | 0.23 | 0.21 | 0.56 | 0.19 | 0.48 | 0.51 | 0.34 | 0.47 | 0.33 |
| Olig | 0.08 | 0.08 | 0.08 | 0.12 | 0.09 | 0.36 | 0.4 | 0.11 | 0.29 | 0.18 |
| microglia | 0.04 | 0.09 | 0.06 | 0.44 | 0.05 | 0.06 | 0.08 | 0.11 | 0.1 | 0.24 |
| endo.mural | 0.1 | 0.09 | 0.1 | 0.11 | 0.1 | 0.14 | 0.16 | 0.11 | 0.29 | 0.17 |
| astrocytes | 0.1 | 0.13 | 0.14 | 0.11 | 0.16 | 0.12 | 0.14 | 0.16 | 0.12 | 0.13 |

**Table S3.** Root mean square error (RMSE) between deconvolution results and ground truth cell type distributions in the seqFISH10000 dataset, including RMSE per cell type and the summed RMSE across all cell types. Values are rounded to two decimal places.

|  | HIDF | RCTD | CARD | Cell2location | Redeconv | Tangram | GraphST | SPACEL | SpatialPrompt | scMoE |
| --- | --- | --- | --- | --- | --- | --- | --- | --- | --- | --- |
| iNeuron | 0.12 | 0.11 | 0.13 | 0.14 | 0.11 | 0.14 | 0.15 | 0.13 | 0.32 | 0.18 |
| eNeuron | 0.16 | 0.21 | 0.2 | 0.56 | 0.17 | 0.48 | 0.49 | 0.34 | 0.46 | 0.33 |
| Olig | 0.08 | 0.08 | 0.09 | 0.12 | 0.1 | 0.36 | 0.36 | 0.1 | 0.27 | 0.18 |
| microglia | 0.04 | 0.09 | 0.06 | 0.46 | 0.05 | 0.06 | 0.07 | 0.1 | 0.16 | 0.16 |
| endo.mural | 0.1 | 0.09 | 0.09 | 0.11 | 0.09 | 0.14 | 0.16 | 0.12 | 0.18 | 0.18 |
| astrocytes | 0.09 | 0.12 | 0.13 | 0.12 | 0.15 | 0.12 | 0.15 | 0.16 | 0.15 | 0.1 |
| Total | 0.58 | 0.7 | 0.71 | 1.49 | 0.66 | 1.31 | 1.39 | 0.96 | 1.55 | 1.12 |

**Table S4.** Root mean square error (RMSE) between deconvolution results and ground truth cell type distributions in the MERFISH100 dataset, including RMSE per cell type and the summed RMSE across all cell types. Values are rounded to two decimal places.

|  | HIDF | RCTD | CARD | Cell2location | Redeconv | Tangram | GraphST | SPACEL | SpatialPrompt | scMoE |
| --- | --- | --- | --- | --- | --- | --- | --- | --- | --- | --- |
| Inhibitory | 0.16 | 0.32 | 0.38 | 0.18 | 0.29 | 0.35 | 0.38 | 0.27 | 0.31 | 0.27 |
| Excitatory | 0.14 | 0.19 | 0.17 | 0.17 | 0.17 | 0.16 | 0.22 | 0.15 | 0.25 | 0.15 |
| OD | 0.09 | 0.19 | 0.23 | 0.08 | 0.11 | 0.28 | 0.4 | 0.08 | 0.13 | 0.1 |
| Microglia | 0.04 | 0.18 | 0.33 | 0.06 | 0.07 | 0.06 | 0.06 | 0.11 | 0.17 | 0.14 |
| Endothelial | 0.07 | 0.11 | 0.17 | 0.06 | 0.09 | 0.08 | 0.13 | 0.1 | 0.17 | 0.09 |
| Astrocyte | 0.09 | 0.11 | 0.11 | 0.06 | 0.15 | 0.1 | 0.13 | 0.11 | 0.13 | 0.08 |
| Total | 0.58 | 1.1 | 1.38 | 0.61 | 0.87 | 1.02 | 1.31 | 0.83 | 1.18 | 0.83 |

**Table S5.** Root mean square error (RMSE) between deconvolution results and ground truth cell type distributions in the MERFISH50 dataset, including RMSE per cell type and the summed RMSE across all cell types. Values are rounded to two decimal places.

|  | HIDF | RCTD | CARD | Cell2location | Redeconv | Tangram | GraphST | SPACEL | SpatialPrompt | scMoE |
| --- | --- | --- | --- | --- | --- | --- | --- | --- | --- | --- |
| Inhibitory | 0.25 | 0.38 | 0.44 | 0.23 | 0.33 | 0.4 | 0.44 | 0.34 | 0.36 | 0.28 |
| Excitatory | 0.21 | 0.27 | 0.24 | 0.23 | 0.22 | 0.23 | 0.29 | 0.21 | 0.29 | 0.19 |
| OD | 0.15 | 0.22 | 0.28 | 0.15 | 0.15 | 0.3 | 0.44 | 0.13 | 0.19 | 0.17 |
| Microglia | 0.08 | 0.17 | 0.3 | 0.09 | 0.11 | 0.09 | 0.1 | 0.12 | 0.14 | 0.16 |
| Endothelial | 0.1 | 0.15 | 0.21 | 0.1 | 0.12 | 0.13 | 0.19 | 0.13 | 0.17 | 0.12 |
| Astrocyte | 0.14 | 0.18 | 0.19 | 0.11 | 0.17 | 0.17 | 0.22 | 0.15 | 0.18 | 0.18 |
| Total | 0.93 | 1.37 | 1.66 | 0.9 | 1.09 | 1.32 | 1.67 | 1.07 | 1.32 | 1.09 |

**Table S6.** Root mean square error (RMSE) between deconvolution results and ground truth cell type distributions in the MERFISH20 dataset, including RMSE per cell type and the summed RMSE across all cell types. Values are rounded to two decimal places.

|  | HIDF | RCTD | CARD | Cell2location | Redeconv | Tangram | GraphST | SPACEL | SpatialPrompt | scMoE |
| --- | --- | --- | --- | --- | --- | --- | --- | --- | --- | --- |
| Inhibitory | 0.37 | 0.43 | 0.4 | 0.34 | 0.41 | 0.51 | 0.52 | 0.43 | 0.45 | 0.37 |
| Excitatory | 0.31 | 0.38 | 0.35 | 0.33 | 0.31 | 0.33 | 0.39 | 0.28 | 0.35 | 0.3 |
| OD | 0.21 | 0.19 | 0.26 | 0.21 | 0.2 | 0.34 | 0.42 | 0.2 | 0.25 | 0.23 |
| Microglia | 0.12 | 0.09 | 0.31 | 0.12 | 0.11 | 0.13 | 0.15 | 0.13 | 0.15 | 0.18 |
| Endothelial | 0.14 | 0.13 | 0.14 | 0.13 | 0.13 | 0.19 | 0.28 | 0.15 | 0.16 | 0.13 |
| Astrocyte | 0.18 | 0.22 | 0.17 | 0.18 | 0.18 | 0.24 | 0.31 | 0.17 | 0.19 | 0.24 |
| Total | 1.33 | 1.44 | 1.64 | 1.3 | 1.34 | 1.75 | 2.08 | 1.37 | 1.56 | 1.46 |

**Table S7.** The running time of all methods on all datasets is in seconds. Some methods cannot run on the corresponding datasets under standard settings, so their running times are not recorded: Redeconv has an excessively long running time when using cell-level deconvolution on real datasets; GraphST and Tangram cannot run on some datasets on our hardware due to the 24GB memory limit, so they are run on CPU.

|  | HIDF | CARD | RCTD | Cell2location | Redeconv | GraphST | Tangram | SPACEL | spatialPrompt | scMoE | reference cell number | query spot number | share gene number |
| --- | --- | --- | --- | --- | --- | --- | --- | --- | --- | --- | --- | --- | --- |
| seqFISH3000 | 9 | 9 | 27 | 398 | 203 | 3 | 1 | 760 | 3 | 11 | 1691 | 71 | 2781 |
| seqFISH6000 | 17 | 9 | 28 | 403 | 254 | 3 | 2 | 767 | 3 | 14 | 1691 | 71 | 5589 |
| seqFISH10000 | 26 | 9 | 29 | 403 | 206 | 3 | 3 | 853 | 4 | 22 | 1691 | 71 | 9277 |
| MERFISH100 | 22 | 11 | 60 | 655 | 13449 | 9 | 2 | 1644 | 3 | 7 | 1691 | 3067 | 131 |
| MERFISH50 | 105 | 143 | 117 | 750 | 58298 | 76 | 10 | 1645 | 4 | 24 | 1691 | 13375 | 131 |
| MERFISH20 | 427 | 455 | 285 | 1042 | 192825 | - | 34 | 1658 | 10 | 57 | 1691 | 44679 | 131 |
| Mouse brain | 3442 | 312 | 4021 | 3111 | - | - | - | 2475 | 33 | 1499 | 116921 | 6050 | 32982 |
| HumanBreastCancer | 708 | 133 | 455 | 2167 | - | 86 | 1641 | 1927 | 14 | 153 | 45647 | 3798 | 13315 |
| MouseThymus | 1133 | 67 | 292 | 2179 | - | 132 | 3024 | 1964 | 20 | 348 | 69813 | 4468 | 4908 |
| HumanDLPFC | 292 | 78 | 2539 | 3035 | - | 41 | 577 | 3208 | 12 | 236 | 13586 | 3639 | 4603 |
| Xenium | 2197 | 987 | 1041 | 1821 | - | - | 312 | 1678 | 15 | 159 | 11712 | 36602 | 12864 |

**Table S8.** Moran's I for the distribution of cortex-associated neurons and dentate gyrus-related cell types estimated by all methods in the mouse anterior and posterior dataset. Values are rounded to two decimal places.

|  | HIDF | CARD | RCTD | Cell2location | Redeconv | GraphST | Tangram | SPACEL | SpatialPrompt | scMoE |
| --- | --- | --- | --- | --- | --- | --- | --- | --- | --- | --- |
| CA1-ProS | 0.85 | 0.54 | 0.77 | 0.68 | 0.4 | 0.93 | 0.47 | 0.65 | 0.79 | 0.63 |
| CA2-IG-FC | 0.66 | 0.47 | 0.49 | 0.6 | 0.52 | 0.89 | 0.17 | 0.47 | 0.51 | 0.56 |
| CA3 | 0.87 | 0.64 | 0.8 | 0.74 | 0.79 | 0.94 | 0.33 | 0.76 | 0.85 | 0.65 |
| DG | 0.81 | 0.6 | 0.74 | 0.75 | 0 | 0.91 | 0.39 | 0.7 | 0.8 | 0.71 |
| L2/3 IT CTX | 0.91 | 0.67 | 0.73 | 0.82 | 0 | 0.92 | 0.4 | 0.74 | 0.92 | 0.55 |
| L4/5 IT CTX | 0.82 | 0.71 | 0.74 | 0.85 | 0.01 | 0.88 | 0.44 | 0.69 | 0.81 | 0.66 |
| L5 IT CTX | 0.95 | 0.72 | 0.83 | 0.91 | 0.88 | 0.86 | 0.24 | 0.83 | 0.93 | 0.38 |
| L5 PT CTX | 0.62 | 0.55 | 0.4 | 0.47 | 0 | 0.87 | 0.12 | 0.49 | 0.64 | 0.67 |
| L5/6 NP CTX | 0.31 | 0.59 | 0.19 | 0.57 | 0.01 | 0.86 | 0.07 | 0.59 | 0.59 | 0.56 |
| L6 CT CTX | 0.88 | 0.73 | 0.69 | 0.83 | 0.04 | 0.93 | 0.38 | 0.78 | 0.73 | 0.32 |
| L6 IT CTX | 0.78 | 0.68 | 0.39 | 0.84 | 0 | 0.88 | 0.11 | 0.6 | 0.78 | 0.45 |
| L6b CTX | 0.67 | 0.66 | 0.37 | 0.4 | 0.12 | 0.84 | 0.13 | 0.41 | 0.54 | 0.57 |

**Table S9.** Spearman's correlation coefficients between estimated cell type abundance (cortex-associated neurons and dentate gyrus-related cells) and their corresponding marker gene expression in the mouse forebrain/hindbrain dataset, calculated across all methods. Values are rounded to two decimal places.

| Cell Type | Gene | HIDF | CARD | RCTD | Cell2location | Redeconv | GraphST | Tangram | SPACEL | SpatialPrompt | scMoE |
| --- | --- | --- | --- | --- | --- | --- | --- | --- | --- | --- | --- |
| CA1-ProS | Wfs1 | 0.25 | 0.29 | 0.32 | 0.44 | 0.04 | 0.08 | 0.11 | 0.23 | 0.22 | -0.04 |
| L2/3 IT CTX | Wfs1 | 0.3 | 0.24 | 0.33 | 0.34 | 0.02 | 0.29 | 0.18 | 0.37 | 0.2 | -0.07 |
| CA2-IG-FC | Rgs14 | 0.3 | 0.2 | 0.25 | 0.37 | 0 | 0.1 | -0.26 | 0.04 | 0.14 | -0.01 |
| CA3 | Nptxr | 0.34 | 0.57 | 0.39 | 0.14 | 0.56 | -0.03 | -0.19 | 0.05 | 0.27 | 0.58 |
| DG | C1ql2 | 0.08 | 0.13 | 0.26 | 0.2 | 0 | 0.28 | 0.21 | 0.15 | 0.04 | -0.19 |
| L2/3 IT CTX | Cux1 | 0.14 | 0.06 | 0.06 | 0.1 | -0.02 | -0.03 | -0.03 | 0.02 | 0.07 | 0.07 |
| L2/3 IT CTX | Cux2 | 0.27 | 0.22 | 0.22 | 0.24 | 0.03 | 0.13 | 0.09 | 0.22 | 0.24 | -0.04 |
| L4/5 IT CTX | Rorb | 0.15 | 0.14 | 0.17 | 0.23 | 0.02 | 0.18 | 0.2 | 0.19 | 0.15 | -0.09 |
| L5 IT CTX | Fezf2 | 0.34 | 0.36 | 0.38 | 0.3 | 0.29 | 0.28 | 0.16 | 0.3 | 0.3 | 0.04 |
| L5 PT CTX | Fezf2 | 0.27 | 0.25 | 0.26 | 0.31 | 0.02 | 0 | 0.08 | 0.23 | 0.2 | -0.25 |
| L4/5 IT CTX | Tbr1 | 0.32 | 0.56 | 0.28 | 0.43 | 0.02 | 0.17 | 0.23 | 0.47 | 0.34 | -0.35 |
| L6 CT CTX | Tbr1 | 0.37 | 0.59 | 0.28 | 0.4 | -0.02 | 0.12 | 0.13 | 0.4 | 0.36 | -0.31 |
| L6 IT CTX | Tbr1 | 0.32 | 0.57 | 0.3 | 0.56 | 0.01 | 0.43 | 0.11 | 0.56 | 0.51 | -0.49 |
| L6 CT CTX | Tle4 | 0.28 | 0.22 | 0.24 | 0.22 | -0.02 | 0.17 | 0.18 | 0.32 | 0.21 | -0.12 |
| L6 IT CTX | Tle4 | 0.25 | 0.22 | 0.19 | 0.24 | -0.01 | 0.18 | 0.05 | 0.27 | 0.23 | -0.12 |
| L6b CTX | Nr4a2 | 0.22 | 0.26 | 0.18 | 0.24 | 0.04 | 0.16 | 0.05 | 0.3 | 0.16 | 0.18 |

**Table S10.** Moran's I for estimated cell type abundance across all methods in the human breast cancer dataset. Values are rounded to two decimal places.

| Cell Type | HIDF | CARD | RCTD | Cell2location | Redeconv | GraphST | Tangram | SPACEL | SpatialPrompt | scMoE |
| --- | --- | --- | --- | --- | --- | --- | --- | --- | --- | --- |
| B cell | 0.76 | 0.38 | 0.52 | 0.5 | 0.5 | 0.75 | 0.26 | 0.52 | 0.78 | 0.72 |
| NK cell | 0.36 | 0.17 | 0.07 | 0.34 | 0.4 | 0.72 | 0.04 | 0.41 | 0.75 | 0.77 |
| T cell | 0.69 | 0.26 | 0.48 | 0.59 | 0.39 | 0.75 | 0.35 | 0.54 | 0.74 | 0.63 |
| fibroblast | 0.77 | 0.56 | 0.6 | 0.57 | 0.46 | 0.69 | 0.44 | 0.54 | 0.83 | 0.51 |
| luminal cell | 0.87 | 0.74 | 0.77 | 0.73 | 0.73 | 0.82 | 0.53 | 0.72 | 0.87 | 0.81 |
| luminal progenitor | 0.66 | 0.16 | 0.48 | 0.47 | 0.7 | 0.84 | 0.27 | 0.51 | 0.85 | 0.66 |
| lymphatic endothelial cell | 0.32 | 0.44 | 0.35 | 0.25 | 0.13 | 0.65 | 0.38 | 0.33 | 0.44 | 0.7 |
| macrophage/DC/monocyte | 0.7 | 0.47 | 0.56 | 0.63 | 0.5 | 0.74 | 0.27 | 0.45 | 0.64 | 0.56 |
| muscle cell | 0.55 | 0.43 | 0.34 | 0.45 | 0.5 | 0.7 | 0.17 | 0.27 | 0.52 | 0.69 |
| myoepithelial cell | 0.47 | 0.39 | 0.3 | 0.36 | 0.56 | 0.76 | 0.17 | 0.55 | 0.51 | 0.73 |
| pDC | 0.15 | 0 | 0.1 | 0.18 | 0.36 | 0.57 | 0.01 | 0.36 | 0.61 | 0.79 |
| plasma cell | 0.83 | 0.69 | 0.63 | 0.58 | 0.62 | 0.65 | 0.15 | 0.57 | 0.65 | 0.7 |
| vascular endothelial cell | 0.4 | 0.31 | 0.35 | 0.39 | 0.23 | 0.64 | 0.3 | 0.36 | 0.67 | 0.39 |

**Table S11.** Spearman's correlation coefficients between estimated abundance of selected cell types and their corresponding marker gene expression in the human breast cancer dataset, calculated across all methods. Values are rounded to two decimal places.

| Cell Type | Gene | HIDF | CARD | RCTD | Cell2location | Redeconv | GraphST | Tangram | SPACEL | SpatialPrompt | | scMoE |
| --- | --- | --- | --- | --- | --- | --- | --- | --- | --- | --- | --- | --- |
| fibroblast | FAP | 0.22 | 0.16 | 0.25 | 0.27 | 0.12 | 0.14 | 0.19 | 0.25 | | 0.11 | 0.3 |
| fibroblast | COL1A2 | 0.8 | 0.58 | 0.82 | 0.82 | 0.43 | 0.54 | 0.66 | 0.77 | | 0.65 | 0.62 |
| fibroblast | VIM | 0.65 | 0.3 | 0.56 | 0.58 | 0.36 | 0.22 | 0.46 | 0.48 | | 0.54 | 0.41 |
| fibroblast | THY1 | 0.44 | 0.21 | 0.42 | 0.44 | 0.25 | 0.25 | 0.32 | 0.37 | | 0.34 | 0.42 |
| fibroblast | S100A4 | 0.3 | 0.04 | 0.25 | 0.28 | 0.19 | 0.1 | 0.2 | 0.17 | | 0.23 | 0.2 |
| luminal cell | GATA3 | 0.6 | 0.54 | 0.6 | 0.6 | 0.59 | 0.54 | 0.5 | 0.6 | | 0.56 | 0.56 |
| luminal cell | FOXA1 | 0.47 | 0.46 | 0.48 | 0.47 | 0.45 | 0.4 | 0.4 | 0.48 | | 0.4 | 0.45 |
| luminal cell | ESR1 | 0.42 | 0.37 | 0.44 | 0.43 | 0.43 | 0.38 | 0.37 | 0.41 | | 0.45 | 0.4 |
| luminal cell | MKI67 | 0.26 | 0.19 | 0.24 | 0.22 | 0.24 | 0.24 | 0.21 | 0.23 | | 0.31 | 0.23 |
| luminal cell | CCND1 | 0.36 | 0.26 | 0.37 | 0.35 | 0.37 | 0.33 | 0.31 | 0.34 | | 0.49 | 0.3 |
| lymphatic endothelial cell | LYVE1 | 0.06 | 0.02 | 0.06 | 0.09 | 0.12 | 0.05 | 0.11 | 0.07 | | 0.04 | 0.08 |
| lymphatic endothelial cell | FLT4 | 0.07 | 0.02 | 0.07 | 0.25 | 0.07 | 0.05 | 0.06 | 0.09 | | 0.04 | -0.01 |
| lymphatic endothelial cell | PROX1 | 0.07 | 0.03 | 0.08 | 0.07 | 0.17 | 0.06 | 0.11 | 0.08 | | 0.06 | 0.06 |
| lymphatic endothelial cell | PDPN | 0.07 | -0.08 | 0.02 | 0.12 | 0.06 | 0.02 | 0 | 0.02 | | -0.01 | -0.07 |
| macrophage/DC/monocyte | PTPRC | 0.37 | 0.04 | 0.31 | 0.38 | 0.27 | -0.01 | 0.14 | 0.24 | | 0.27 | 0.3 |
| macrophage/DC/monocyte | ITGAM | 0.16 | 0.1 | 0.16 | 0.15 | 0.14 | 0.09 | 0.15 | 0.19 | | 0.07 | 0.2 |
| muscle cell | ACTA2 | 0.32 | 0.29 | 0.25 | 0.38 | 0.28 | 0.33 | 0.23 | 0.21 | | 0.26 | 0.18 |
| muscle cell | TAGLN | 0.41 | 0.31 | 0.21 | 0.42 | 0.36 | 0.5 | 0.23 | 0.19 | | 0.37 | 0.25 |
| muscle cell | MYLK | 0.09 | 0.06 | 0.06 | 0.15 | 0.1 | 0.08 | 0.06 | 0.03 | | 0.08 | -0.01 |
| muscle cell | MYH11 | 0.15 | 0.12 | 0.17 | 0.17 | 0.14 | 0.14 | 0.14 | 0.15 | | 0.1 | 0.1 |
| myoepithelial cell | KRT5 | 0.08 | 0 | 0.18 | 0.17 | 0.07 | 0.27 | 0.19 | 0.23 | | 0.15 | 0.08 |
| myoepithelial cell | KRT14 | 0.1 | -0.03 | 0.19 | 0.17 | 0.05 | 0.23 | 0.16 | 0.22 | | 0.12 | 0.06 |
| myoepithelial cell | KRT17 | 0.08 | -0.03 | 0.2 | 0.15 | 0.04 | 0.26 | 0.16 | 0.22 | | 0.12 | 0.07 |
| vascular endothelial cell | PECAM1 | 0.34 | 0.17 | 0.29 | 0.34 | 0.19 | 0.21 | 0.2 | 0.25 | | 0.19 | 0.26 |
| vascular endothelial cell | KDR | 0.11 | 0.11 | 0.15 | 0.17 | 0 | 0.09 | 0.1 | 0.12 | | 0.01 | 0.03 |
| B-plasma cell | IGHA1 | 0.53 | 0.6 | 0.56 | 0.59 | 0.57 | 0.29 | 0.07 | 0.54 | | 0.38 | 0.45 |
| B-plasma cell | IGHM | 0.49 | 0.49 | 0.54 | 0.54 | 0.52 | 0.3 | 0.1 | 0.53 | | 0.44 | 0.43 |
| B-plasma cell | IGHA2 | 0.07 | 0.13 | 0.18 | 0.13 | 0.12 | 0.12 | 0.14 | 0.12 | | 0.1 | 0.16 |
| B-plasma cell | PRDM1 | 0.03 | 0.03 | 0.02 | 0.08 | 0.07 | 0.03 | 0.03 | 0.02 | | 0.05 | 0.07 |
| B-plasma cell | CD38 | 0.06 | 0.09 | 0.06 | 0.12 | 0.13 | 0.1 | 0.08 | 0.09 | | 0.12 | 0.14 |
| B-plasma cell | IRF4 | 0.09 | 0.13 | 0.12 | 0.14 | 0.14 | 0.11 | 0.1 | 0.12 | | 0.12 | 0.15 |
| T cell | CD4 | 0.24 | -0.05 | 0.18 | 0.27 | 0.05 | 0.12 | 0.01 | 0.19 | | 0.17 | -0.03 |
| T cell | CD8A | 0.18 | 0.04 | 0.24 | 0.24 | 0.06 | 0.16 | 0.08 | 0.21 | | 0.2 | -0.05 |
| T cell | CD8B | 0.16 | 0.02 | 0.21 | 0.2 | 0.05 | 0.12 | 0.09 | 0.18 | | 0.15 | 0.01 |
| NK cell | CD3D | 0.26 | -0.03 | -0.03 | 0.29 | 0.07 | 0.19 | 0.06 | 0.08 | | 0.17 | -0.07 |
| NK cell | GNLY | 0.28 | 0.01 | 0.35 | 0.35 | 0.09 | 0.17 | 0.11 | 0.24 | | 0.17 | -0.03 |
| NK cell | KLRD1 | 0.18 | 0.02 | 0.16 | 0.22 | 0.06 | 0.13 | 0.08 | 0.16 | | 0.16 | -0.01 |

**Table S12.** Moran's I for the spatial distribution of T-cell abundance at distinct thymocyte different stages of differentiation estimated by all methods in the mouse thymus dataset. Values are rounded to two decimal places.

|  | HIDF | CARD | RCTD | Cell2location | Redeconv | GraphST | Tangram | SPACEL | SpatialPrompt | scMoE |
| --- | --- | --- | --- | --- | --- | --- | --- | --- | --- | --- |
| DN | 0.47 | 0.28 | 0.22 | 0.36 | 0.06 | 0.73 | 0.26 | 0.34 | 0.6 | 0.41 |
| DP | 0.84 | 0.63 | 0.71 | 0.79 | 0.73 | 0.86 | 0.44 | 0.68 | 0.83 | 0.75 |
| Mature T | 0.71 | 0.45 | 0.31 | 0.81 | 0 | 0.86 | 0.26 | 0.4 | 0.7 | 0.53 |
| Immature T | 0.4 | 0.14 | 0.14 | 0.51 | 0.13 | 0.77 | 0.17 | 0.46 | 0.55 | 0.45 |
| Treg | 0.75 | 0.53 | 0.29 | 0.81 | 0 | 0.87 | 0.2 | 0.57 | 0.84 | 0.61 |

**Table S13.** Spearman's correlation coefficients between estimated abundance of DN, DP, and immature T cells and their corresponding marker gene expression in the mouse thymus dataset, calculated across all methods. Values are rounded to two decimal places.

| Cell Type | Gene | HIDF | CARD | RCTD | Cell2location | Redeconv | GraphST | Tangram | SPACEL | SpatialPrompt | scMoE |
| --- | --- | --- | --- | --- | --- | --- | --- | --- | --- | --- | --- |
| DN | Il2ra | 0.18 | 0.1 | 0.3 | 0.23 | -0.01 | 0.22 | 0.06 | 0.17 | 0.14 | -0.06 |
| DP | Cd4 | 0.23 | 0.17 | 0.21 | 0.17 | 0.15 | 0.17 | 0.19 | 0.22 | 0.21 | 0.22 |
| DP | Cd8a | 0.3 | 0.3 | 0.3 | 0.28 | 0.23 | 0.25 | 0.28 | 0.34 | 0.27 | 0.33 |
| DP | Cd8b1 | 0.39 | 0.38 | 0.37 | 0.34 | 0.29 | 0.35 | 0.33 | 0.42 | 0.35 | 0.41 |
| Immature T | Cd24a | -0.02 | -0.01 | -0.06 | 0.08 | -0.01 | -0.13 | 0.03 | -0.11 | -0.05 | -0.14 |
| Immature T | Mki67 | 0.04 | -0.13 | -0.03 | -0.01 | -0.04 | -0.38 | 0.01 | -0.18 | -0.07 | -0.35 |
| Treg | Ikzf2 | 0.11 | 0.14 | 0.14 | 0.08 | -0.03 | 0 | 0.02 | 0.09 | -0.02 | -0.01 |
| Treg | Tnfrsf4 | 0.23 | 0.2 | 0.18 | 0.24 | -0.03 | 0.26 | 0.18 | 0.23 | 0.23 | 0.21 |
| Treg | Tnfrsf18 | 0.14 | 0.14 | 0.08 | 0.12 | 0 | 0.1 | 0.08 | 0.1 | 0.12 | 0.06 |

**Table S14.** Moran's I for estimated cell type abundance across all methods in the human DLFPC dataset. Values are rounded to two decimal places.

|  | HIDF | CARD | RCTD | Cell2location | Redeconv | GraphST | Tangram | SPACEL | SpatialPrompt | scMoE |
| --- | --- | --- | --- | --- | --- | --- | --- | --- | --- | --- |
| L2/3 IT | 0.94 | 0.34 | 0.45 | 0.63 | 0.16 | 0.69 | 0.04 | 0.55 | 0.89 | 0.51 |
| L4 | 0.81 | 0.31 | 0.4 | 0.3 | 0 | 0.77 | 0 | 0.41 | 0.68 | 0.71 |
| L5 IT | 0.97 | 0.41 | 0.54 | 0.56 | 0.4 | 0.76 | 0.08 | 0.45 | 0.78 | 0.66 |
| L5 PT | 0.82 | 0.3 | 0.15 | 0.29 | 0.08 | 0.56 | 0.01 | 0.3 | 0.55 | 0.28 |
| L6 CT | 0.83 | 0.29 | 0.11 | 0.21 | 0.03 | 0.8 | 0.05 | 0.46 | 0.68 | 0.32 |
| L6 IT | 0.81 | 0.2 | 0.21 | 0.21 | 0.11 | 0.77 | 0.03 | 0.31 | 0.78 | 0.55 |
| L6b | 0.54 | 0.07 | 0.07 | 0.13 | 0.44 | 0.78 | 0.04 | 0.25 | 0.54 | 0.46 |
| Oligo | 0.96 | 0.88 | 0.93 | 0.91 | 0.91 | 0.97 | 0.75 | 0.92 | 0.96 | 0.91 |

**Table S15.** The point-biserial correlation coefficients between neuronal subtypes and cortical layers in the human DLPFC dataset. Values are rounded to two decimal places.

| Cell Type | Domain | HIDF | CARD | RCTD | Redeconv | Cell2location | GraphST | Tangram | SPACEL | SpatialPrompt | scMoE |
| --- | --- | --- | --- | --- | --- | --- | --- | --- | --- | --- | --- |
| L2/3 IT | Layer2-Layer3 | 0.7 | 0.46 | 0.55 | 0.12 | 0.62 | 0.49 | 0.17 | 0.6 | 0.6 | -0.05 |
| L4 | Layer3 | 0.51 | 0.29 | 0.3 | -0.02 | 0.28 | 0.63 | 0.08 | 0.34 | 0.59 | 0.43 |
| L5 IT | Layer4-Layer5 | 0.78 | 0.53 | 0.6 | 0.2 | 0.62 | 0.58 | 0.17 | 0.5 | 0.73 | 0.59 |
| L5 PT | Layer3 | 0.68 | 0.36 | 0.3 | 0.11 | 0.36 | 0.13 | 0.05 | 0.33 | 0.47 | 0.21 |
| L6 CT | Layer6 | 0.67 | 0.31 | 0.23 | -0.03 | 0.39 | 0 | 0.13 | 0.19 | 0.55 | 0.4 |
| L6 IT | Layer6 | 0.62 | 0.11 | 0.27 | -0.05 | 0.3 | 0.63 | 0.07 | 0.26 | 0.5 | 0.04 |
| L6b | Layer6 | 0.41 | 0.13 | 0.17 | 0.25 | 0.22 | 0.65 | 0.15 | 0.33 | 0.38 | 0.07 |
| Oligo | WM | 0.91 | 0.88 | 0.89 | 0.89 | 0.88 | 0.85 | 0.8 | 0.9 | 0.9 | 0.88 |

**Table S16.** Spearman's correlation coefficients between estimated abundance of neuronal subtypes and their corresponding marker gene expression in the Xenium dataset, calculated across all methods. Values are rounded to two decimal places.

| Cell Type | Gene | HIDF | CARD | RCTD | Cell2location | Redeconv | GraphST | Tangram | SPACEL | SpatialPrompt | scMoE |
| --- | --- | --- | --- | --- | --- | --- | --- | --- | --- | --- | --- |
| CA1-ProS | Wfs1 | 0.19 | 0.05 | 0.16 | 0.34 | 0.12 | 0.01 | 0.03 | 0.21 | 0.2 | 0.26 |
| L2/3 IT CTX | Wfs1 | 0.25 | 0.16 | 0.25 | 0.2 | 0.18 | 0.28 | 0.25 | 0.23 | 0.2 | 0.26 |
| L2/3 IT CTX | Cux2 | 0.29 | 0.43 | 0.51 | 0.53 | 0.25 | 0.24 | 0.2 | 0.53 | 0.44 | 0.5 |
| L4/5 IT CTX | Rorb | 0.36 | 0.35 | 0.4 | 0.38 | 0.25 | 0.28 | 0.31 | 0.34 | 0.28 | 0.5 |
| DG | Prox1 | 0.43 | 0.45 | 0.58 | 0.4 | 0.23 | 0.4 | 0.3 | 0.54 | 0.09 | 0.6 |
| L6 CT CTX | Tle4 | 0.23 | 0.18 | 0.24 | 0.2 | 0.2 | 0.26 | 0.24 | 0.22 | 0.18 | 0.26 |

**Table S17: Summary of method attributes**

|  | **Model Complexity** | **Computational Cost** | **Interpretability** | **Input Requirements** | **GPU support** |
| --- | --- | --- | --- | --- | --- |
| HIDF | Variable, Adaptive,  Θ(NB) per mini-batch iteration | Meidum | Flexible  & Hierachy-aware | Standardized input requirements | Yes |
| CARD | Low,Ω(TM) complexity | Low | Fixed |  | No |
| RCTD | Low,Ω(TM) complexity | High | Fixed |  | No |
| Cell2location | Low,Ω(TM) complexity | High | Fixed |  | Yes |
| Redeconv | High,Ω(NM) complexity | High | Flexible |  | No |
| GraphST | High,Ω(NM) complexity | High | Weak |  | Yes |
| Tangram | High,Ω(NM) complexity | High | Flexible |  | Yes |
| SPACEL | Variable, Architecture-Dependent | High | Weak |  | Yes |
| SpatialPrompt | Variable, Implementation-Dependent | Low | Weak |  | No |
| scMoE | Variable, Architecture-Dependent | Low | Fixed | Prior-Dependent | Yes |

| Dataset | Spots | Genes | Species | Download |
| --- | --- | --- | --- | --- |
| MERFISH 100 | 3067 | 135 | Mus musculus | https://zenodo.org/records/7674290 |
| MERFISH 50 | 13375 | 135 |  |  |
| MERFISH 20 | 44679 | 135 |  |  |
| seqFISH 3000 | 71 | 3000 |  |  |
| seqFISH 6000 | 71 | 6000 |  |  |
| seqFISH 10000 | 71 | 10000 |  |  |
| Mouse anterior-posterior brain | 6050 | 32285 |  | https://zenodo.org/records/6925603 |
| Mouse thymus | 4468 | 17987 |  | https://zenodo.org/records/10362607 |
| Mouse brain Xenium | 36602 | 248 |  | https://www.10xgenomics.com/datasets/xenium-human-lung-cancer-post-xenium-technote |
| Human breast cancer | 3798 | 36601 | Homo sapiens | https://zenodo.org/records/6925603 |
| Human DLPFC 151673 | 3639 | 33538 |  |  |

**Table S18.** Relevant information and download path of spatial transcriptomic datasets.

| Dataset | Cells | Genes | Species | Download |
| --- | --- | --- | --- | --- |
| scRNA-seq reference for MERFISH and seqFISH simulated datasets | 1691 | 19972 | Mus musculus | https://zenodo.org/records/7674290 |
| Mouse Whole Cortex and Hippocampus 10x | 116921 | 22764 |  | https://portal.brain-map.org/atlases-and-data/rnaseq/mouse-whole-cortex-and-hippocampus-10x |
| Mouse thymus | 72042 | 5125 |  | https://zenodo.org/records/12583813 |
| Mouse brain GSE115746 | 13586 | 41754 |  | <https://www.ncbi.nlm.nih.gov/geo/query/acc.cgi?acc=>  GSE115746 |
| Human breast cancer | 45647 | 5000 | Homo sapiens | https://zenodo.org/records/6925603 |

**Table S19.** Relevant information and download path of scRNA-seq reference datasets.
